# The effect of manuscript characteristics on quality assessment of scientific manuscripts in psychology

**DOI:** 10.1371/journal.pone.0355938

**Published:** 2026-09-15

**Authors:** Hilde E.M. Augusteijn, Jelte M. Wicherts, Klaas Sijtsma, Marcel A.L.M. van Assen

**Affiliations:** 1 Department Methodology and Statistics, Tilburg University, the Netherlands; 2 Department of Sociology, Utrecht University, the Netherlands; Institute of Medical Biochemistry Leopoldo de Meis (IBqM) - Federal University of Rio de Janeiro (UFRJ), BRAZIL

## Abstract

We report a vignette study and a survey to investigate which study characteristics influence quality ratings psychology academics give of articles submitted for publication, and psychology academics and students to theses written by psychology students. In the vignette study, 800 respondents evaluated the quality of an abstract (7-point scale) of studies with small (*N* = 54) or large sample sizes (*N* = 540), showing statistically significant or non-significant results, and containing statistical reporting errors or no errors. In the subsequent survey, the same participants rated the importance of 29 manuscript characteristics related to a study’s theory, design, conduct, data analyses, and presentation for assessing either the quality of a manuscript or its publishability (article) or grade (thesis). Results showed that quality ratings were affected by sample sizes but not by statistical significance or the presence of statistical reporting errors in the rated research vignette. Furthermore, in the survey academics and students provided highly similar ratings of the importance of different aspects relevant to quality assessment of articles and theses. These results suggest that quality criteria for scientific manuscripts are already adopted by students and are similar for submitted manuscripts and theses. Further research is needed to examine which study characteristics researchers consider most important when assessing a manuscript’s quality in peer review.

## Introduction

Publication bias is widespread in many research areas. In psychology, over 90% of the published papers report a statistically significant effect [1–3], but the average power of studies is estimated well below .50 [4–7]. This high prevalence of statistically significant results is inconsistent with the small effects that are often studied using underpowered designs [8]. The sources of publication bias are twofold. On the one hand, journals generally prefer publishing articles with statistically significant results [9,10]. Journal editors prefer articles with nice stories based on novel and statistically significant results and tend to ignore articles with unexpected null results and many ifs and buts [11]. On the other hand, authors themselves cause publication bias [12–14]; they are less likely to submit results that are not statistically significant. Perhaps authors already anticipate possible rejection or lose interest in their project when their expectations are not met. Either way, studies with statistically non-significant results are more likely to end up in the so-called file drawer, producing a biased representation of a research topic or area based on only the significant results [15].

To avoid the file-drawer effect and resulting publication bias, authors have an incentive to increase the probability of finding a statistically significant result. They can increase the probability while designing their study but also after data collection has been completed. The ‘correct’ way of improving one’s chances if a true nonzero effect exists is to increase statistical power using larger samples or by using appropriate methodology (e.g., meta-analysis [16]) or statistical techniques [17]. However, due to practical or financial constraints, it is often difficult for researchers to increase their sample sizes. Another, albeit questionable way to increase the probability of finding a statistically significant result, is the opportunistic use of so-called researcher degrees of freedom [18]. Researchers can use these degrees of freedom to increase type I error [19] and statistical power by analyzing their data in various ways, decide to stop data collection after looking at the results, drop conditions from analyses, or remove outliers. These manipulations are also known as *p*-hacking, which is an example of questionable research practices (QRPs). Unfortunately, many psychology researchers have admitted having engaged in QRPs [20–24]. QRPs and publication bias are considered important causes of irreproducible findings in psychological research [25].

Peer review is a gatekeeper that is expected to protect scientific literature against publication bias and QRPs and to guarantee that the published scientific studies meet a minimum level of quality. Given that much of the published literature does not seem to meet the high-quality criteria we desire, and published results often fail to replicate [(e.g., 25, 26)], the current study examines which manuscript characteristics influence peer reviewers’ quality assessment of scientific manuscripts, including students’ master’s theses. We also examine which manuscript characteristics are assumed to be important for quality assessment by scientists and students. We first discuss what is currently known about quality assessment in peer-review and education before presenting our research questions and hypotheses.

### Peer review

Following Kelly et al. [27, p. 227], we define peer review as “a process of subjecting an author’s scholarly work, research or ideas to the scrutiny of others who are experts in the same field”. The peer review process has been a formal part of scientific communication since the first scientific journals were published over 300 years ago [28]. Although there are differences between journals, peer review typically entails the next steps: (a) an editor makes an initial manuscript selection by checking journal fit and whether a submitted manuscript meets the formal standards of, for example, format and ethics; (b) the editor selects and invites reviewers; (c) the reviewers independently assess the quality of the work and make recommendations concerning publication; and (d) if the editor decides the manuscript is a candidate for publication, she guides the process of resubmission and additional rounds of review and decides upon definitive publication.

The first goal of peer review is to check whether reported research meets the appropriate standards, and therefore whether findings and conclusions can be considered valid. The second goal is to help authors improve the quality of their research and its presentation. The third goal is to assess originality, significance, and broader interest, and finally, to assess the ‘fit’ between a paper and a journal [29].

Despite its ubiquitous use, peer review is intensely debated. Ware [30] found that most academics (85%) believe that peer review greatly helps scientific communication. In contrast, others argued that peer review might increase the use of QRPs due to reviewers demanding ‘perfect’ results that are unrealistic [11,31], and/or researchers expecting reviewers to demand overly clean results [24]. It may well be that critical peer reviewers stimulate, either implicitly or perhaps sometimes even explicitly, authors to leave out unconvincing outcomes or aspects of the study [22,24] or even to misreport certain results. Hence, the quality of reviews remains a concern and little consensus exists on how to even define review quality [32,33].

It is unclear to what extent peer review helps improve research manuscripts. Peer review seems to bring about little change in manuscripts from preprint to post-print [32], which does not need to imply that the original research was flawless. Fraud and QRPs still enter the published literature, and published articles are retracted from publication after peer review failed to detect problems in these articles [27,32,33]. Furthermore, experimental studies have shown that reviewers have a hard time detecting even obvious errors in a paper [34–36]. Although most authors and reviewers believe that the detection of plagiarism is a task of peer review, a minority believes peer review is suited to do so [27].

Reviewers are often inconsistent and agree only slightly above chance in their opinion on whether a paper should be published [37,38]. Inter-reviewer reliability is low [39,40], with quality ratings of the same manuscript ranging from unacceptable to excellent [33,41]. Whereas one reviewer argues ‘I found this paper an extremely muddled paper with a large number of deficits’, another reviewer argues ‘It is written in a clear style and would be understood by any reader’ [38]. Assuming almost all reviewers are honest and are not abusing their power of peer review (e.g., to stall publication of competitors or scoop their ideas), these inconsistencies indicate that the evaluation of manuscript quality is ambiguous. Every peer reviewer has a different background and different knowledge, and editors might be looking for peer reviewers with differing expertise to review an article. While this is a sound strategy using different reviewers’ complementary expertise, unanticipated differences in knowledge and understanding of the same topic, knowledge of previous studies on the same topic, methodological and statistical skills, and personal preferences for some topics but not for others may readily produce inconsistent review assessments. Differences between reviewers in knowledge, understanding, and preferences are difficult to control in research. Therefore, in this study, we focus on the question of which manuscript characteristics are most important for manuscript quality rating that might differ between reviewers and even between manuscripts.

Previous research indicates that reviewers seem to favor their judgements of scientific manuscripts on ‘false cues’, such as statistical significance, often unduly complex procedures, and overly complex writing [42]. Furthermore, reviewers suffer from the same biases as researchers, such as confirmation bias and hind-sight bias [43,44]. They might not even realize these false cues influence their quality assessments. For example, in an experimental study by Atkinson, et al. [45], peer reviewers received a version of a manuscript with either statistically significant results (*p* < .01), results with *p* < .10, or results with *p* < .25. The manuscript that reported a statistically significant result was three times less likely to be rejected than the other two versions. Despite all other characteristics of the manuscript being equal, reviewers often believed they rejected the manuscript based on the design of the study rather than its results.

It is unclear if reviewers differ in their assessment of the quality of a manuscript or whether they assess the suitability of the manuscript for publication. Due to (perceived) expectations of journals and editors, perhaps other characteristics are important for assessing publishability of an article. Statistical significance may be considered more relevant for publishability than for manuscript quality. Reviewers may recognize high quality research but use different characteristics when assessing its ‘publishability’.

### Review in education

There are other review issues relevant in assessing a master’s thesis than in reviewing scientific manuscripts as part of the publication process. With their master’s thesis, students show what they have learned during their education. We would expect teachers to positively consider cues that indicate responsible research practices (RRPs), but to negatively assess indications of QRPs. Since the main goal of a master’s thesis is educational, that is, the application of knowledge, insights, and skills previously learned, but usually not publication, statistical significance of the results should be less important than in manuscripts submitted for publication. In addition, students do not believe that there is a causal relation between good science and statistically significant results, and they do not believe their teachers reward statistically significant results [46]. If students’ perspective is correct, we expect academics to uphold different quality standards for master’s theses than for research manuscripts. Furthermore, theses differ from published manuscripts in many aspects that may be relevant for their review and evaluation. For example, previous research showed that theses report effect sizes more frequently than published manuscripts [46–48], on average use larger samples [49], and conduct a priori power analysis more often [46,50]. These differences could also indicate that students and researchers consider different aspects in assessing the quality of these manuscripts.

Students are often confronted with published literature as examples of good research reports during their education. These examples, as well as students’ perceived attitude of their academic teachers towards QRPs shape the students’ own attitudes and behavior towards these QRPs [46] and set the bar for what is considered high-quality research. For example, what students believe may be a sufficient sample size (e.g., to have sufficient statistical power) might be influenced by sample sizes encountered in the literature studied in various courses, and when studying in a field where open science is advocated in the published literature, students might assign greater importance to pre-registration and sharing study materials. Previous research shows that both researchers and students frequently engage in QRPs. Over 90% of psychology researchers admitted having engaged in QRPs during their career [20,21,23,51]. Estimates for students indicate that 64% of them have already engaged in QRPs in their short academic careers (up to graduate level, [52]), whereas 40% of psychology students engaged in QRPs in their thesis [46]. It is currently unknown how academics differ in their quality assessment of theses and articles, and how potential QRPs affect this assessment. Finally, it is unknown how students perceive this quality assessment of their supervisors.

### Current study

We know little about the characteristics of submitted manuscripts and master’s theses that affect quality assessments and their publishability or grades. It also is unknown whether students and their supervisors believe quality is affected by the same characteristics. Therefore, the main research question of this study is “Which characteristics of a manuscript affect students and academic researchers when assessing the quality of a scientific manuscript?”. We note that the preregistered research question was originally formulated as: “Which characteristics of a manuscript do students and researcher believe to be of importance when assessing the quality of a scientific manuscript?”. This formulation was changed, because we believed, in hindsight, that this formulation did not cover the full range of our study. We did not only investigate the beliefs of academics and students, but also tested what influenced their quality assessment in the vignette study that we preregistered together with the survey study.

In our vignette study, we focused on three research characteristics that we manipulated in a study’s abstract to examine their effect on the quality evaluation of scientific manuscripts (article or thesis). We studied the impact of sample size, statistical significance, and a statistical reporting error on quality ratings of the research. A statistical reporting error refers to an inconsistency between the reported test statistic, degrees of freedom, and *p*-value. Furthermore, we studied the two-way interactions of these three characteristics, and the effect of career phase (student versus academic) and manuscript type (thesis versus article) on the ratings of the research.

The following research questions and matching hypotheses were formulated and preregistered (https://osf.io/cd3uw):

RQ1: Which characteristics of research (sample size, significance, reporting error) as described in a manuscript (thesis or article) affect the quality assessment of a scientific manuscript by both students and academics?

H1a: Manuscripts with large sample size are rated as having higher quality compared to manuscripts with smaller sample sizes

H1b: Manuscripts with significant results are rated as having higher quality compared to manuscripts with non-significant results

H1c: Manuscripts with reporting errors are rated the same as manuscripts without reporting errors

RQ2: Do the effects of the characteristics on the quality of the manuscript as assessed by academics differ between a thesis and an article?

H2a: The differences in quality rating between small and large sample size manuscripts is larger for articles compared to theses

H2b: The differences in quality rating between significant and non-significant manuscripts is larger for articles compared to theses

H2c: The differences in quality rating between manuscript with and without reporting error do not differ for articles compared to theses

RQ3: Do the effects of the characteristics on the quality of a thesis differ between students and academics?

H3a: The differences in quality rating between small and large sample size manuscripts differs between students and academics

H3b: The differences in quality rating between significant and non-significant manuscripts is larger for students compared to academics

H3c: The differences in quality rating between manuscripts with and without reporting error does not differ between students and academics

**Fig 1:**
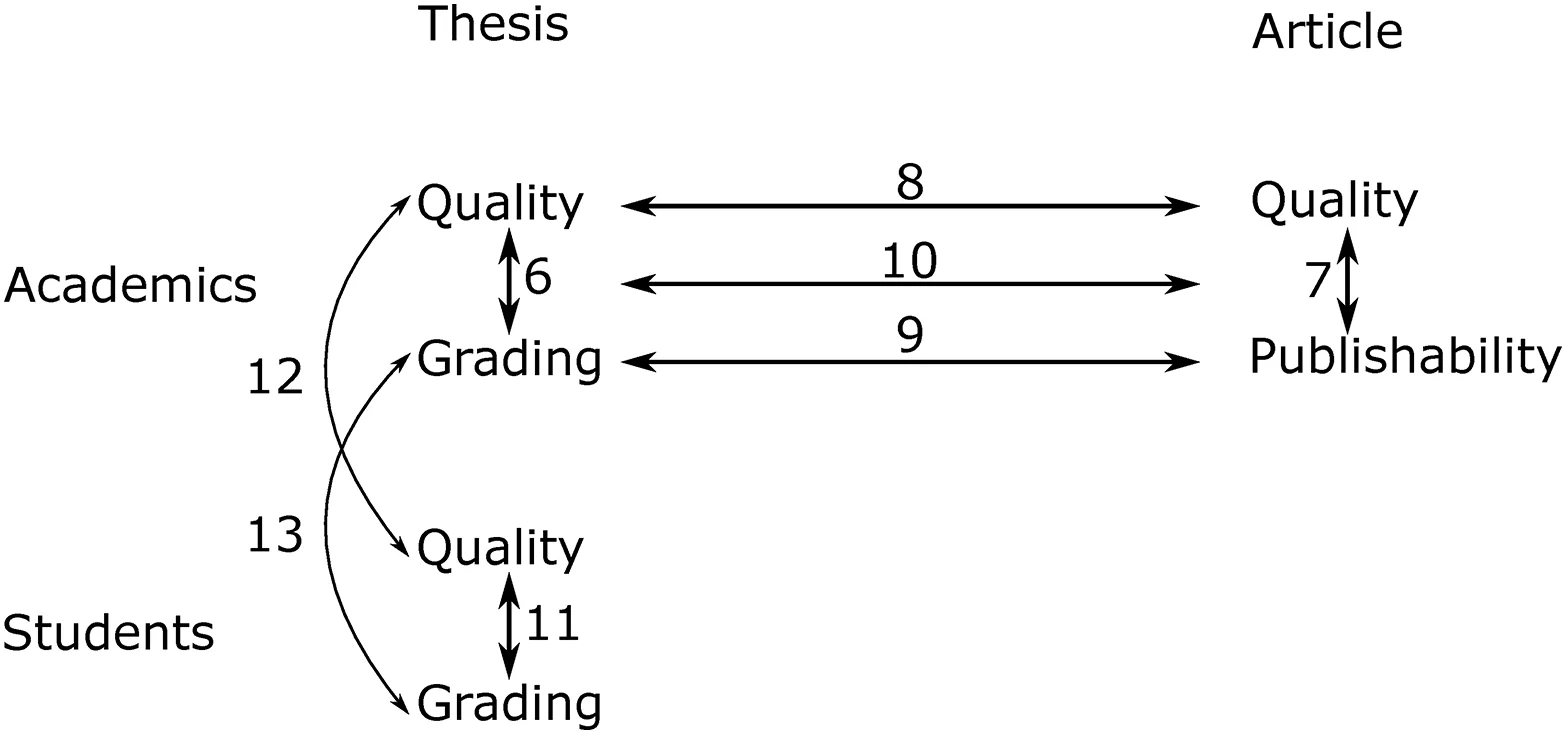
Overview of Explorative Research Questions of the Survey Study on the Evaluation of Scientific Manuscripts. Six different evaluations are made. Academics (top) evaluate both ‘quality’ and ‘grading’ of theses (left) and the ‘quality’ and ‘publishability’ of articles (right). Students (bottom) only evaluate the ‘quality’ and ‘grading’ of’theses’ (left). Arrows 6-9 and 11-13 refer to correlations between two evaluations. For instance, and more specifically, arrow 9 corresponds to the correlation between the evaluated importance of 29 manuscript characteristics for ‘grading theses by academics’ and the evaluated importance of these same manuscript characteristics for the ‘publishability of articles by academics’. Arrow 10 refers to a difference between two correlations. The preregistration lists all research questions verbally.

We explored the importance of a broader set of manuscript characteristics that could be relevant for the evaluation of manuscript quality. We formulated no a priori hypotheses for these characteristics but considered a set of research questions to compare different conditions. First, we studied which characteristics were considered relevant when assessing the quality and the grading of a thesis (RQ4) and when assessing the quality and the publishability of an article (RQ5). For academics, we studied the differences in the relevance of the different characteristics when assessing quality and when assessing publishability/grade for both theses (RQ6) and articles (RQ7), differences between the importance of characteristics when assessing quality of theses versus articles (RQ8), differences between the importance of characteristics when assessing the grade or publishability of theses versus articles (RQ9), and differences between the differences of the importance of characteristics when assessing quality versus grade/publishability of theses versus articles (RQ10). For students, we studied differences in the relevance they believe their supervisors assign to characteristics when assessing quality versus grade (RQ11). Finally, we studied differences in relevance of characteristics between students and academics when assessing quality of thesis (RQ12) and grades (RQ13). Fig 1 shows research questions 6–13 and their relations.

## Method

### Design

The pre-registered project (https://osf.io/cd3uw) consists of a vignette study and a survey. Conditions varied across six design factors. First, participants were either students or academics (factor: career phase). Second, participants were asked about the evaluation of either theses (academics and students) or articles submitted for publication (academics only) (factor: manuscript type). The third, fourth and fifth factors pertained to only the vignette part of the study. The text of the vignette was an abstract describing a study with either a small or a large sample size, main results were either statistically significant or not, and the vignette either contained a statistical reporting error or not. The sixth factor only pertained to the survey part of the study. Participants of the vignette were subsequently asked in the survey to evaluate the importance of a list of manuscript characteristics related to either (i) quality assessment of an article/thesis, or (ii) publishability of an article or a thesis grading (factor: quality assessment type). In total, there were 48 conditions (for academics: 2 manuscript type × 2 sample size × 2 statistical significance × 2 reporting error × 2 quality assessment type = 32. For students: 2 sample size × 2 statistical significance × 2 reporting error × 2 quality assessment type = 16).

### Power analysis

To determine the sample size needed to evaluate the hypotheses, we conducted an a priori power analysis. Since we only formulated hypotheses for research questions 1–3, we based our power analysis on these hypotheses. Based on a fixed-effect ANOVA with 24 conditions and the effect of a factor with two levels (df = 1), desiring a power of .8 with α = .05, and assuming a small effect size *f* = .10, we needed at least 787 participants. Assuming 33 participants per condition, this meant 528 academics and 264 students.

### Participants

Data were collected among psychology students and academics who had published within the field of psychology. Exclusion criteria for students were not studying psychology or having finished one’s education program. For academics, exclusion criteria were having no experience with grading theses (in thesis condition) or having no experience with peer reviewing an article (in article condition). PhD candidates were considered as academics rather than students. The sampling strategy between academics and students differed substantially, and we therefore describe their data collection procedures separately.

**Fig 2:**
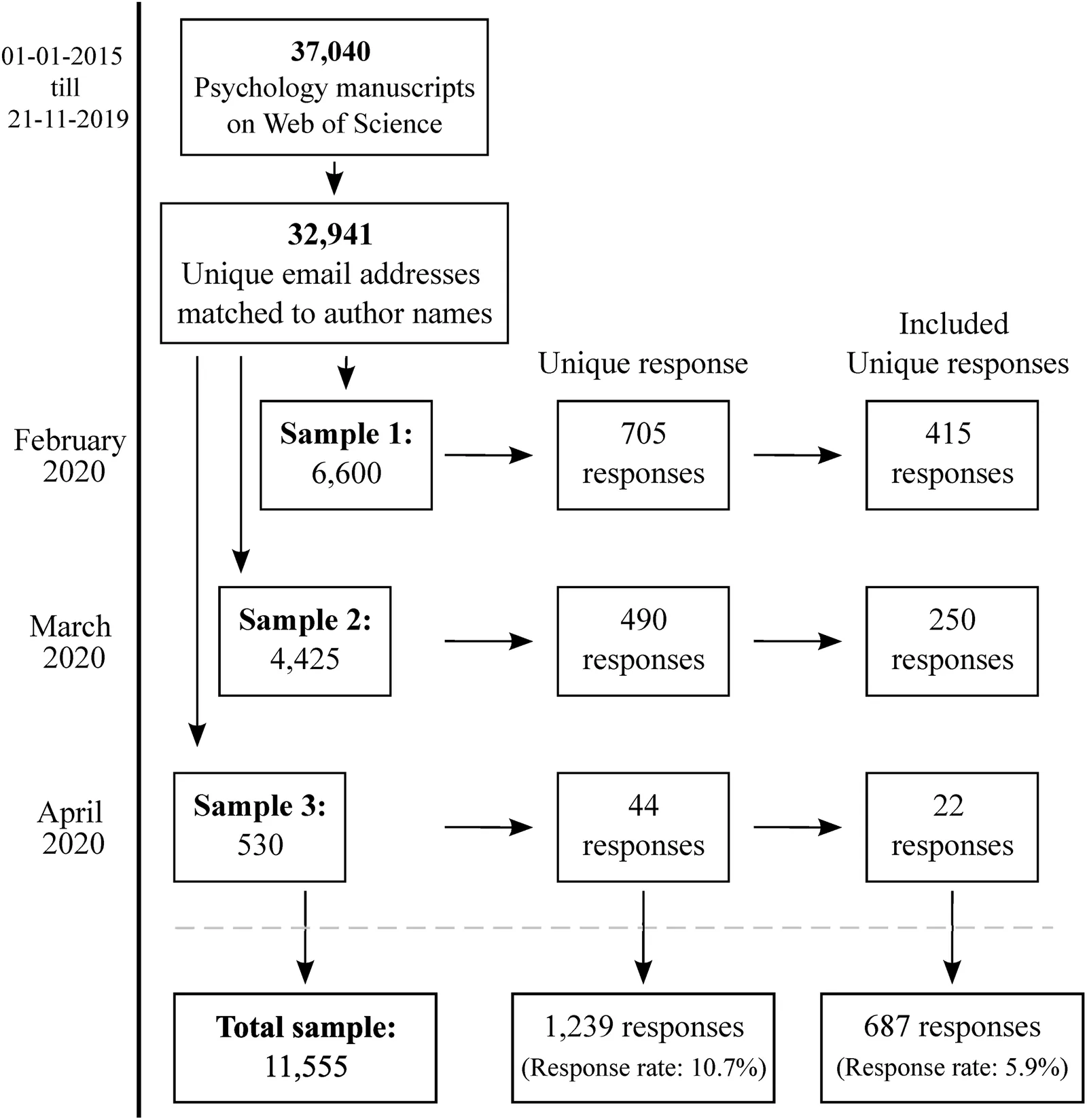
Overview of the Sampling Procedure Amongst Academics.

In November 2019, contact information of academics who published articles or editorial materials in psychology was extracted from Web of Science. E-mail addresses were automatically matched to the author names and academics received a personalized invitation to complete the survey. Three rounds of sampling were needed to generate enough responses. Data collection took place between February 2020 and April 2020. In these three rounds, a total of 11,555 authors and editors was contacted, and 1,239 of them started the survey (response rate of 10.7%). However, many of them did not complete the survey (and were removed from the dataset) or were excluded from the sample due to the exclusion criteria. A total of 687 academics provided responses that were included in the analyses (response rate 5.9%). Fig 2 provides an overview of the sampling procedure of academics. More detailed information can be found on OSF (https://osf.io/j2gpk/). Participants were randomly assigned to one of the 32 academics conditions.

The student survey was distributed via social media, as well as through the professional network of the primary researchers and their direct colleagues. An invitation to participate was placed on Twitter, as well as 51 international psychology student Facebook groups. Unfortunately, Facebook flagged the invitation as spam and removed all messages. In a second attempt, moderators from 49 large psychology student Facebook groups were asked to post a message with the survey invitation in their Facebook groups. 24 groups replied positively and either posted the message themselves or allowed us to post the message and pinned it to the top of the Facebook group so it would be noticeable. These Facebook groups had almost 65,000 members in total. The survey was also posted on two survey sharing Facebook groups, with an additional 40,000 international psychology students.

Despite the effort, response rate was very low amongst international psychology students. After 11 months, data collection was closed after 301 students participated in the survey. Participants were randomly allocated to the 16 student-conditions. Unfortunately, 188 of the 301 participants needed to be excluded from analysis, because they did not complete the survey (160), did not study psychology [20], or had recently graduated from their studies or worked already as a PhD candidate [8]. The final number of student responses included was 113. Due to both the low number of responses and the subsequent low statistical power to detect small effect sizes, all hypotheses and analyses that include students (Research questions 3, 11, 12 and 13, see Fig 1) should be considered exploratory instead of confirmatory.

### Participant characteristics

Researchers and students included in our sample originated from all continents. Academics were in different stages of their career and worked in various research fields (see Table 1 for participant characteristics). Many researchers (256, 37.2%) indicated they did not work in any of the pre-specified research areas, even though these participants had published on topics within the pre-specified area of psychology. Deviating from our preregistration, we decided not to remove participants from other fields, thereby retaining the planned statistical power for our analyses (note: tests of hypotheses H1a-c to H3a-c excluding those participants yielded the same substantive conclusions, see https://osf.io/84ugb).

**Table 1:** Characteristics of Participating Researchers and Students.

		Researchers (n = 687)	Students (n = 113)
Continent			
	Europe	338 (49.2%)	
	Europe, the Netherlands		55 (49.1%)
	Europe, other		20 (17.9%)
	North America	222 (32.3%)	8 (7.1%)
	South America	35 (5.1%)	3 (2.7%)
	Africa	9 (1.3%)	12 (10.7%)
	Asia	47 (6.8%)	11 (9.8%)
	Australia & Oceania	36 (5.2%)	3 (2.7%)
	Missing		1 (0.9%)
Career phase			
	PhD	77 (11.2%)	
	PostDoc	55 (8.0%)	
	Researcher	46 (6.7%)	
	Teacher	10 (1.5%)	
	Assistant Professor	100 (14.6%)	
	Associate Professor	157 (22.9%)	
	Full Professor	200 (29.1%)	
	Other	42 (6.1%)	3 (2.7%)
	Bachelor		73 (64.6%)
	Master		37 (32.7%)
Research Field			
	Applied Psychology	74 (10.8%)	
	Biological Psychology	10 (1.5%)	
	Clinical Psychology	45 (6.6%)	
	Developmental Psychology	37 (5.4%)	
	Educational Psychology	49 (7.1%)	
	Experimental Psychology	52 (7.6%)	
	Mathematical Psychology	15 (2.2%)	
	Multidisciplinary Psychology	33 (4.8%)	
	Psychoanalysis	2 (0.3%)	
	Social Psychology	114 (16.6%)	
	Other	256 (37.3%)	

### Ethical review and data management

This research project received an exemption for ethical review by the ethical review board of Tilburg University (ERB, EC-2019.EX135) due to anonymous data collection with minimal risk since the obtained information (a) cannot be traced directly or indirectly to the individual, and (b) does not harm or discomfort the individual in any way. Informed consents were also reviewed by the ERB. Informed consent was obtained by all participants at the start of the survey, by confirming to participate by clicking a button. The data management plan has been approved by the data representatives at Tilburg University. The data management plan describes data storage, open data plans, a pre-DPIA (Data protection Impact Assessment) and a description of GDPR agreements and compliance. Both are available from the preregistration on OSF (https://osf.io/cd3uw).

### Procedure

All data were collected using Qualtrics. Participants first answered a set of general questions. Students were asked whether they studied psychology, where they studied (continent), in which phase of their study they were (bachelor/undergraduate, master/graduate, other, namely...), and whether they had ever written a thesis (bachelors’ and/or masters’ thesis). Academics in the thesis condition were asked if they had ever graded a thesis (bachelor/undergraduate level, master/graduate level, PhD level), and in the peer-reviewed manuscript condition they were asked whether they had ever peer reviewed an article submitted for publication. Furthermore, all academics were asked where they worked (continent), what position they held, and in which psychology research field they primarily worked. Categorization was based on the psychology subfields as distinguished in the Clarivate Analytics Master Journal List (https://mjl.clarivate.com). If participants indicated that they did not study psychology (student conditions), had never graded a thesis (academic-thesis condition), or had never peer reviewed a paper (academics-article condition), they were thanked for their participation and redirected to the end of the questionnaire.

### Vignette study

Participants were asked to read an abstract of a manuscript that was submitted for publication (academics), or an abstract of a master’s thesis (students and academics). The text of the abstract was written by the principal investigators, and its face validity was assessed by a clinical psychologist in the field of personality psychology (see Box 1). Three characteristics of the abstract were manipulated; sample size (small or large; italics: N = 54 or N = 540), statistical significance of the main finding (yes or no; underlined: *p* = .005 or *p* = .6), and reporting error (yes or no; bold; *p* < .001 (incorrect) or *p* = .01 (correct)).

**Box 1. Text of Vignette Study with Manipulated Characteristics Sample size (Italics), Statistical Result (Bold), and Reporting Error (Underlined).**

**Abstract**

**Background:** Test anxiety can have a large impact on a student’s academic career. Previous research shows higher anxiety, as well as higher neuroticism and conscientiousness, for women than for men. We examined whether sex differences in neuroticism and conscientiousness can explain possible sex differences in test anxiety.

**Methods:** In this experimental study, [*540/ 54*] participants ([*300/ 30*] male and [*240/ 24*] female) filled out the Mowen’s Personality Scale and were randomly assigned to one of two conditions. In the high-stakes condition, participants watched a video clip and completed a fast-paced 10-item test about what they had seen (memory task); payment depended on recall performance, and participants knew they were graded relative to other participants. In the low-stakes (control) condition, participants watched the same video clip but answered ten unrelated questions; participants only received a show-up fee. All participants were asked to report anxiety (using the Spielberger State-Trait Anxiety Inventory) twice: once before randomization (pre-test) and again just before completing the ten items described above (post-test).

**Results:** The increase in anxiety (i.e., post-test – pre-test) between the two conditions was, in line with previous research, higher for women than for men ([***t*(536) = 2.33, *p* < .001**/ ***t*(50) = 2.40, *p* < .001**/ ***t*(536) = 2.33, *p* = .01**/ ***t*(50) = 2.40, *p =* .01**]). [Contrary to/ Confirming] our hypothesis, sex differences in neuroticism and conscientiousness could [not] explain the sex difference in increased anxiety ([*F*(4,532) = 0.689, *p* = .6/ *F*(4,46) = 0.694, *p* = .6/ *F*(4,532) = 3.76, *p* = 0.005/ *F*(4,46) = 4.28, *p* = .005]).

**Conclusions:** Sex differences in test anxiety are [not/ at least partially] explained by sex differences in neuroticism and conscientiousness.

Based on the abstract, participants were asked to rate the quality of the master’s thesis/scientific manuscript on a 7-point scale ranging from very low quality to very high quality. Next, they were asked to name three aspects of this thesis/manuscript that were most relevant for their assessment of its quality.

### Survey

The questionnaire asked participants how important different manuscript characteristics are when assessing the (i) quality and (ii) grading of a thesis (by students and academics), and the (iii) quality and (iv) publishability of an article (only by academics). The instructions of each condition are provided in Table 2.

**Table 2:** Formulation of Survey Instruction for all six Conditions.

Student condition – thesis Quality	Student Condition – thesis Grading	Researcher Condition – thesis Quality	Researcher Condition – thesis Grading	Researcher condition – article Quality	Researcher Condition – article Publishability
“In the following questions, you will be asked to indicate the extent to which you believe different characteristics of the study/report are important when...					
supervisors assess the quality of a master’s thesis.”	supervisors determine the grade of a master’s thesis.”	assessing the quality of a master’s thesis.”	determining the grade of a master’s thesis.”	assessing the quality of a submitted manuscript.”	assessing a manuscript’s suitability for publication in a peer-reviewed journal.”

After these instructions, participants were asked to rate the importance of 29 manuscript characteristics (see Table 3) on a 7-point scale ranging from ‘Not at all important’ to ‘Of the utmost importance’. These 29 characteristics were related to different aspects of a manuscript: theory, design, conduct, (data) analysis, and presentation. All 29 characteristics were presented in a random order different for different participants.

**Table 3:** Manuscript Characteristics Evaluated by the Participating Researchers and Students.

Category	Abr.	Manuscript characteristic
Theory	T1	Describing the relevant theories.
	T2	Deriving hypotheses from theory.
	T3	Explicitly stating the hypotheses to be tested.
	T4	Using a large number of literature references.
	T5	Clearly describing the study objective.
Design	D1	Using an appropriate study design.
	D2	Clearly describing the sample population.
	D3	Reporting pre-study calculation of sample size.
	D4	Having a large sample size.
	D5	Using a complex design.
	D6	Pre-registering (part of) the study.
Conduct	C1	Achieving a high response rate.
	C2	Achieving high statistical power.
	C3	Applying complex statistical analyses.
	C4	Providing open data (i.e., making data available online).
	C5	Providing open research materials (i.e., making the materials available online or in the manuscript).
	C6	Providing open analysis code (i.e., making the code available online or in the manuscript).
Analysis & presentation	A1	Adequately describing all used statistical procedures.
	A2	Using the appropriate statistical analyses.
	A3	Distinguishing confirmatory from exploratory statistical analyses.
	A4	Reporting assumption checks.
	A5	Clearly presenting the statistical results.
	A6	Providing confidence intervals for the main statistical results.
	A7	Providing effect sizes for the main statistical results.
	A8	Reporting the statistical results without errors.
	A9	Observing large effect sizes.
	A10	Observing the main effect in the hypothesized direction.
	A11	Reaching statistical significance of the results of the main hypothesis.
	A12	Drawing the correct conclusion from the statistical analysis.

### Statistical analysis

Data were analyzed using R (version 4.1.1). In the preregistration, an analysis plan was described, and R-code was included to answer research questions 1–3, as well as R-code for a planned principal component analysis (PCA) that could be used to prepare data for research questions 4–13 (https://osf.io/fn5tu/). Research questions (RQ) 1–3 were answered using linear regression analyses, by testing the main effects of manuscript characteristics (sample size (H1a), statistical significance of the effect (H1b), reporting error (H1c)); by adding to the model and testing the three interactions manuscript characteristic × manuscript type (thesis vs. article) (H2a-H2c); and by adding and testing the three interactions manuscript characteristic × actor (student vs. academic (H3a-H3c) (see preregistration for the equations).

As the outcome variables are Likert scales with seven ordered response categories, we also analyzed the data using quantile (median) regression (Quantreg R-package, version 5.86, [53]) as a sensitivity analysis. For the confirmative analyses (vignette study, hypothesis 1–3), the alpha level was set to.05.

Since hypotheses 1c, 2c and 3c indicated no effect and null hypothesis significance testing cannot provide support for null hypotheses we evaluated these hypotheses using Bayesian statistics. We computed posterior model probabilities for four models ($L\left(data|\mu_{i}\right)$), assuming a zero ($\mu_{\text{0}}$), small ($\mu_{s}$), medium ($\mu_{m}$), or large effect ($\mu_{l}$), respectively, all having the same prior probability (uniform prior distribution). The relative posterior model probabilities of the effect size (zero, small, medium, large) were computed as


$$\begin{array}{c} \text{Pr}\left(\mu_{i}|data\right)=\;\frac{L\left(data|\mu_{i}\right)}{L\left(data|\mu_{\text{0}}\right)+\;L\left(data|\mu_{s}\right)+L\left(data|\mu_{m}\right)+L\left(data|\mu_{l}\right)}, \end{array}$$

with *L* being the likelihood of the observed *F*-value, given a zero, small, medium or large effect, using the (non-central) *F*-distribution. The observed *F*-value is the *F*-statistic of the test comparing the regression model with the parameter to be estimated (a simple effect of ‘reporting error’, or the main effect in case the interaction was not statistically significant) to the regression model without that parameter. *L* is distributed as *F*(1,*N*-*k*-1,λ_i_), with *k* the number of predictors in the model (*k* = 3 for H1c, *k* = 6 if the interaction is significant, *k* = 5 if the interaction is not included in the model for H2c and H3c), and non-centrality parameter λ_i_ = 0, 0.02×(*N-k*-1), 0.15×(*N-k*-1), 0.35×(*N-k*-1) for a zero, small, medium, large effect, respectively (see OSF, https://osf.io/sk5t7/, for R-code). We interpreted a relative posterior probability of at least .75 for a zero true effect-size (corresponding to a Bayes Factor of 3 or more) as evidence in favor of the null-hypothesis (i.e., a zero true effect-size).

The aspects the respondents named in the open-ended question that followed the vignette study (“Please name the three aspects of this manuscript that were most relevant for your assessment of its quality”) were analyzed using content analysis. The aspects named by participants were categorized by the principal investigator (HA). Categories emerged from the data itself, and specific categories with less than five mentions were put in the ‘other category’.

To analyze the importance of the 29 manuscript characteristics in the survey, a PCA was planned for each of the six survey conditions separately to reduce the dimensionality of the dataset if (i) the Kaiser–Meyer–Olkin (KMO) test indicated sufficiently correlated items (KMO > .6 in all six conditions) and at the same time (ii) items loaded similarly on the principal components across the six conditions. If either of these two criteria were not met, analyses would take place at the item-level and the four intended categories (theory, design, conduct and analysis, and presentation). More details can be found in the preregistration.

The statistical analysis of research questions 4–13 was exploratory and was not part of the preregistration. To investigate these research questions, we provide descriptive statistics and correlations between the importance ratings of either the new principal components (RQ 4–5), or their four intended categories (Table 3), of the conditions compared in research questions 6–13 (see also Fig 1). For these exploratory analyses, as a (partial) correction for multiple testing, the alpha level was equal to .01 instead of .05. This provides us with insight in the characteristics most relevant in the quality assessment, according to each group (academics vs students, articles vs theses, quality vs grading/publishability), and the correlations inform us to what extent these groups (dis)agree in their importance ratings.

Both the data and the codebook of the data can be found at osf.io/j2gpk.

## Results

### Vignette study

The mean quality rating of the research manuscript abstract (Box 1) for all participants was 3.84 (sd=1.42). Students on average provided higher quality ratings than academics (4.42 versus 3.75, t(156.02)=4.92, p<.001, d=0.499), and academics on average provided higher quality ratings to the thesis abstracts than to the scientific manuscript abstracts (3.96 versus 3.55, t(678.01)=3.83, p<.001, d=0.292). Table 4 provides mean quality ratings, standard deviations, number of participants in each of the 18 conditions of the vignette study, and effect sizes (Cohen’s *d*) of differences between two conditions.

**Table 4:** Mean Quality Ratings of Scientific Manuscripts for all Conditions and Effect Sizes Comparing Conditions.

		Students (113)	Academics (687)		Comparison	
		Thesis (113)	Thesis (344)	Article (343)	Thesis: Stud-academ.	Academics: thesis-article
Sample size	Small	4.31 (1.40, 62)	3.82 (1.47, 166)	3.43 (1.31, 171)	*d* = 0.343 (*p* = .023)	*d* = 0.281 (*p* = .010)
	Large	4.57 (1.25, 51)	4.08 (1.45, 178)	3.67 (1.32, 172)	*d* = 0.373 (*p* = .021)_	*d* = 0.299 (*p* = .005)
		*d* = 0.199	*d* = 0.181	*d* = 0.183		
Significant	Nonsig	4.55 (1.12, 71)	4.01 (1.48, 173)	3.54 (1.28, 169)	*d* = 0.440 (*p* = .002)	*d* = 0.334 (*p* = .002)
	Sig	4.21 (1.63, 42)	3.91 (1.46, 171)	3.55 (1.36, 174)	*d* = 0.193 (*p* = .268)	*d* = 0.251 (*p* = .020)
		*d* = − 0.229	*d* = − 0.068	*d* = 0.006		
Reporting	Present	4.52 (1.37, 54)	3.95 (1.50, 169)	3.53 (1.30, 170)	*d* = 0.408 (*p* = .011)	*d* = 0.298 (*p* = .006)
Error	Absent	4.34 (1.31, 59)	3.97 (1.44, 175)	3.57 (1.35, 173)	*d* = 0.278 (*p* = .068)	*d* = 0.286 (*p* = .008)
		*d* = −0.134 (*p* = .479)	*d* = 0.013 (*p* = .905)	*d* = 0.028 (*p* = .795)		

*Note.* Standard deviations and sample sizes for each condition between brackets. The penultimate column shows effect sizes of the difference between the quality rating of the thesis abstract by students and academics. The final column shows effect size of the difference between thesis and article abstracts by academics.

Below each design factor (sample size, significance, and reporting error) in Table 4, the effect size of the design factor within a condition is provided. None of these factors influenced the quality ratings within a group of respondents and had generally small effect sizes. Effect sizes for small versus large sample size manuscripts differed from d=0.181 to d=0.199. The effect sizes for manuscripts with statistically significant versus non-significant results differed from d=−0.006 through d=0.228. The presence versus absence of a reporting error resulted in effect sizes between d=−0.028 and d=0.134.

We conducted a linear regression analysis using all respondents (academics and students) to answer the three research questions of hypothesis 1. Sample size, statistical significance, and reporting error were entered as predictor variables to study their impact on the quality rating of the abstract. Only sample size (H1a) predicted quality rating (B=−0.25, SE=0.10, 95% CI:[−0.44, −0.05], t=−2.46,  p=.014), suggesting that small sample sizes received lower quality ratings. Statistical significance (H1b; (B=0.14, SE=0.10, 95% CI:[−0.06, 0.34], t=1.39,  p=.166) and reporting errors (H1c; (B=0.002, SE=0.10, 95% CI:[−0.19, 0.20], t=0.02,  p=.983) did not predict quality rating. The omnibus test, however, indicated that the model with these three predictors together did not predict quality rating, with only 1% of the variance of quality rating explained (95% CI:[.00, .02],F(3, 796)=2.57,  p=.053). Exploratively, we also tested the sample size × statistical significance interaction, but found no effect either (*B* = 0.17, *t* = 0.85, *p* = .398). For hypothesis 1c, relative Bayesian posterior model probabilities were.9996,.0004, < .0001 and <.0001 for null, small, medium, and large effects, respectively, indicating strong evidence in favor of H1c (*BF*_*01*_ = 2,826): that is, there is no effect of reporting errors on the quality ratings of the abstract. Given the high response among academics, we also provide results for academics only: Sample size: *B* = −0.26, *SE* = 0.11, *t* = −2.44, *p* = .015. Statistical significance: *B* = 0.06, *SE* = 0.11, *t* = 0.56, *p* = .575, reporting error: *B* = −0.03, *SE* = 0.11, *t* = −0.249, *p* = .803. The model with the three predictors explained 0.9% of the variance (95% CI: [.00,.02], *F*(3,683) = 2.08, *p* = .10). Relative Bayesian Posterior Model probabilities (H1c): .9984, .0016, < .0001 and <.0001 for a null, small, medium, and large effect respectively (*BF_01_* = 629)

All 800 participants were asked to name three aspects they considered relevant for their evaluation of manuscript quality. The 800 × 3 = 2,400 named aspects were classified in 17 categories. Sometimes all three aspects named by the same respondent were related to one category (e.g., study design), and sometimes one aspect was related to multiple categories (e.g., ‘Clear description of methodology’ is related to both writing and study design). Therefore, the number of categorized answers was not equal to 2,400. The minimum number of differently categorized answers by a participant was 0 (due to incorrect understanding of the question), and the maximum number of different categories was 4. This resulted in a total of 1,976 categorized answers.

Table 5 shows the numbers of respondents naming an aspect related to each category, ranked by prevalence. Aspects related to sample size or power were mentioned by more than 25% of participants. Aspects related to statistical significance were named by only 14 respondents (1.75%) but 68 different respondents (8.5%) named the ‘results’ as an important aspect. Consequently, the maximum number of participants indicating that they were influenced by the strength of the results was 82 (10.25%). Statistical reporting error was mentioned by 13 participants (1.63%). The actual statistical reporting error was identified in only 3 cases (0.38%). Whereas sample size and statistical significance were likely to be commented upon in both the small/large sample size and the significant/non-significant result condition, mentioning (in)correct reporting was only likely for the 393 participants (49.13% of total sample) in the reporting error condition.

**Table 5:** Prevalence of Categories Indicated as Relevant for Assessing the Quality of the Abstract of Scientific Manuscripts and Students’ Theses (Text in Box 1), Provided by Students and Academics.

	Prevalence
Methodology/Research Design	69.25% (n = 554)
Writing (language, style, structure)	35.13% (n = 281)
Sample size/Statistical power	25.13% (n = 201)
Conclusions/interpretation of results	21.50% (n = 180)
Analysis (appropriate, missing tests, etc.)	21.75% (n = 174)
Relevance (novelty, impact, importance, etc.)	17.00% (n = 136)
Theory/Background	14.00% (n = 112)
Reporting of statistical results (e.g., effect size missing)	13.75% (n = 110)
Research question/hypothesis	9.88% (n = 79)
Results	8.50% (n = 68)
Sampling	4.00% (n = 32)
Statistical significance	1.75% (n = 14)
Statistical reporting error (correct or incorrect)	1.63% (n = 13)
Ethics	1.38% (n = 11)
Other (e.g., preregistration, open data)	1.38% (n = 11)

We ran a second regression analysis to investigate the interaction between the manipulated characteristics and manuscript type (thesis versus article, H2). Since students only received the thesis condition, only academics were included in this analysis. The regression model with only the four main predictors (sample size, significance, reporting error and manuscript type) explained 2.95% of the variance (F(4, 682)=5.19, p=.0004). The regression model adding the three two-way interaction terms between sample size, significance, and manuscript type ($R^{\text{2}}=\text{2}.\text{9}\text{9}\%,\;F(\text{7},\;\text{6}\text{7}\text{9})=\text{2}.\text{9}\text{9},\;p=.\text{0}\text{0}\text{4})$, shown on the left panel of Table 6, did not significantly improve the model, it explained only 0.04% more variance (F(3, 679)=0.10, p=.96). Because none of the interactions added to the explanation of quality rating, we cannot support hypothesis 2a or 2b; that is, sample size and statistical significance did not differentially affect quality rating of thesis versus articles for academics. To answer hypothesis 2c, Bayesian relative posterior model probabilities showed that an interaction effect of reporting error and manuscript type is not likely, confirming hypothesis 2c (Bayesian Posterior model probabilities: no effect: .9989, small effect: .0011, medium effect: < .0001, large effect: < .0001; *BF*_*01*_ = 869).

**Table 6:** Results of Regression Analyses Predicting Quality Ratings of Scientific Manuscript with Interactions of Manuscript Type and Career Phase.

	*B (SE)*	*t (p)*	95%CI	Fit [95% CI]	*B (SE)*	*t (p)*	95%CI	Fit [95% CI]
Intercept	3.68 (.15)	24.96 (<.001)	[3.39, 3.97]		4.04 (.15)	26.94 (<.001)	[3.75, 4.34]	
Sample	−0.24 (.15)	−1.60 (.111)	[-0.54, 0.06]		−0.27 (.15)	−1.74 (.083)	[-0.57, 0.04]	
Sig	0.00 (.15)	0.003 (.998)	[-0.30, 0.30]		0.11 (.15)	0.72 (.474)	[-0.19, 0.42]	
Error	−0.03 (.15)	−0.20 (.839)	[-0.33, 0.27]		−0.02 (.15)	−0.14 (.890)	[-0.33, 0.28]	
Manuscript type (MT)	0.36 (.21)	1.72 (.085)	[-0.05, 0.77]					
Sample×MT	−0.03 (.21)	−0.13 (.894)	[-0.45, 0.39]					
Sig × MT	0.11 (.21)	0.52 (.604)	[-0.31, 0.53]					
Error×MT	0.01(.21)	0.04 (.965)	[-0.41, 0.43]					
				*R*^*2*^ = .0299				
				[.00,.05]				
Career phase (CP)					0.17 (.34)	0.49 (.622)	[-0.50, 0.84]	
Sample×CP					−0.02 (.31)	−0.05 (.958)	[-0.63, 0.60]	
Sig × CP					0.28 (.32)	0.85 (.396)	[-0.36, 0.91]	
Error× CP					0.29 (.32)	0.93 (.355)	[-0.33, 0.91]	
								*R*^*2*^ = .034
								[.00,.06]

*Note.* Left side: Regression coefficients of sample size, statistical significance and reporting error and their interactions with manuscript type (MT, reference category is articles) on quality rating, academics only. Right side: regression coefficients of sample size, statistical significance and reporting error and their interactions with career phase (CP, reference category is academics) on quality rating, thesis only.

Our third regression analysis investigated the interaction effects of the three manipulated design factors and career phase (student versus academic, H3; Sample×CP, Sig × CP, Error×CP). The regression model with only main effects explained 3.11% of the variance in quality ratings (F(4, 452)=3.63, p=.006), whereas the model containing both main effects and interactions explained 3.41% of variance in the quality ratings (F(7, 449)=2.27, p=.028), 0.3% more (F(3, 449)=0.47, p=.71). Interactions did not improve the prediction, see right the panel of Table 6. That is, neither sample size (H3a) nor statistical significance (H3b) showed an interaction with career phase in the quality ratings of theses. Bayesian posterior model probabilities supported the hypothesis that there was no interaction between reporting errors and career phase (H3c) since the null model received a probability of.9166 (small effect:.0834, medium effect: < .0001, large effect: < .0001; *BF*_*01*_ = 11).

We ran a preregistered exploratory regression analysis on the full model predicting quality ratings of the manuscript, including all interaction terms up to four-way interactions. This full model explained 7.2% of the variance (F(23, 776)=2.61, p<.001), but of the predictors only sample size had an effect (B=0.69,p=.02). This full model was not beneficial compared to the model with only main effects (F(18, 776)=0.81, p=.68). A quantile regression as robustness check also showed no effects for H1 (neither for both academics and students, nor for academics only), but it did show an interaction effect between sample size and type of manuscript (H2a, *p* = .004). No effects for hypothesis 3 were found. Detailed results of the quantile regression can be found on OSF (https://osf.io/zpk67/). To summarize, we only found a weak effect of sample size on the quality rating of abstracts.

### Survey Study

**Fig 3:**
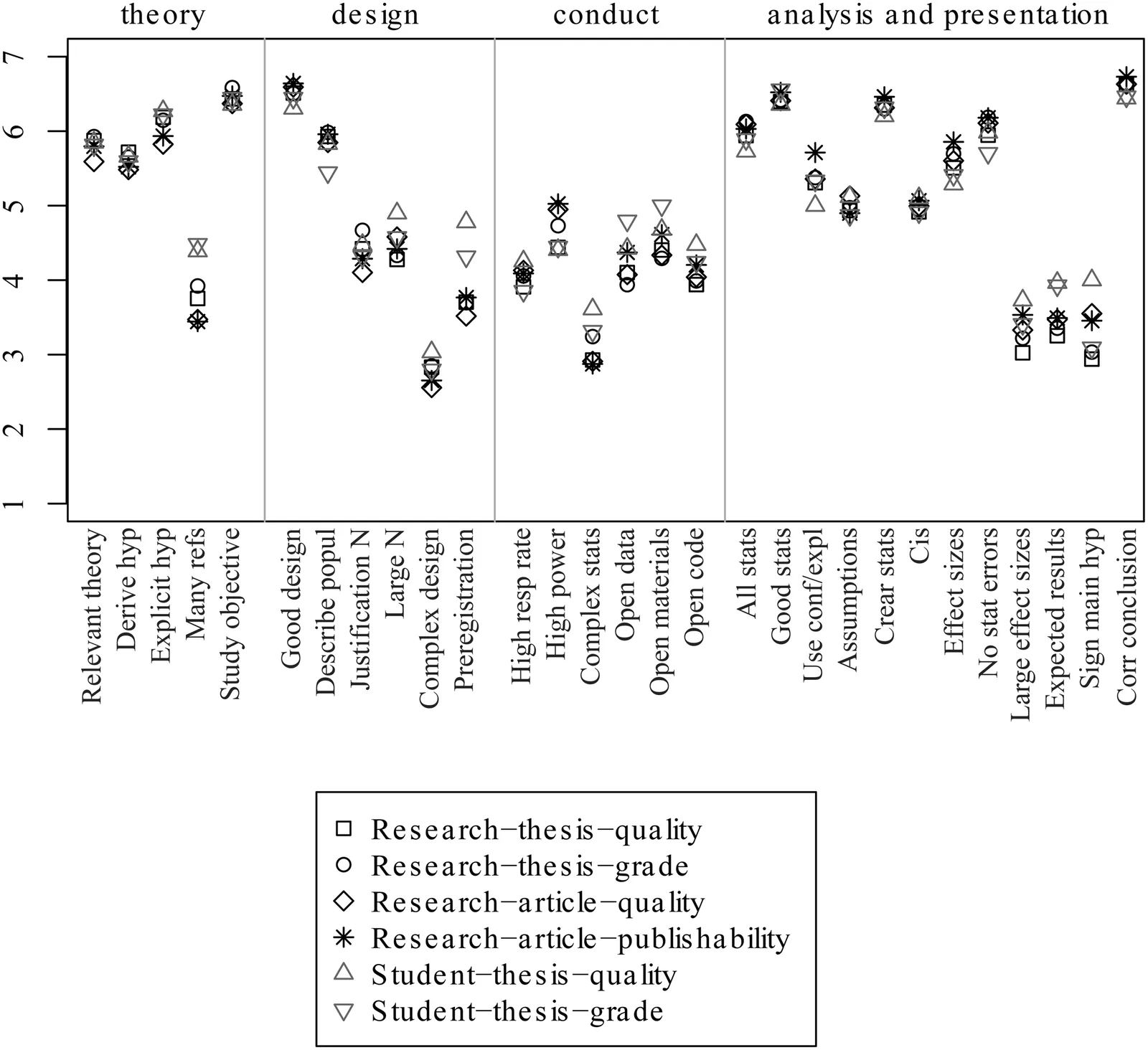
Mean Ratings of all Importance Ratings of Manuscript Characteristics for Quality Assessment for all six Conditions (see legend).

As the items in the survey did not correlate sufficiently (KMO-indices varying from 0.28 to 0.91) in all six conditions (Academics: Thesis quality, Thesis grading, Article quality, Article publishability. Students: Thesis quality, Thesis grading), we analyzed the data at the item-level for each condition separately to answer research questions 4 and 5 (see Table 7 and Fig 3). Table 7 shows the mean rating of each manuscript characteristic in each of the six conditions, as well as the average rating of each category (theory, design, conduct and analysis and presentation). Fig 3 also shows the importance rating for all 29 manuscript characteristics for all six conditions. The order of average importance of the four categories was the same for all six conditions.

**Table 7:** Mean Importance Ratings [1–7] of Manuscript Characteristics and Categories for Quality Assessment, in all Six Conditions (Students-Thesis, Quality & Grading; Academics-Thesis, Quality & Grading; Academics-Article, Quality & Publishability) (Standard Deviations Between Brackets).

	Students		Academics				Total
	Thesis		Thesis		Article		
	Quality	Grading	Quality	Grading	Quality	Publ.	
Describing the relevant theories (T1)	5.85 (1.06)	5.81 (1.08)	5.87 (1.27)	5.93 (1.16)	5.59 (1.22)	5.80 (1.13)	5.80 (1.18)
Deriving hypotheses from theory (T2)	5.66 (1.32)	5.59 (1.30)	5.72 (1.28)	5.65 (1.47)	5.48 (1.24)	5.52 (1.42)	5.60 (1.35)
Explicitly stating the hypotheses to the tested (T3)	6.27 (1.20)	6.22 (1.16)	6.19 (1.09)	6.15 (1.28)	5.82 (1.29)	5.93 (1.16)	6.05 (1.21)
Using a large number of literature references (T4)	4.39 (1.41)	4.48 (1.33)	3.75 (1.45)	3.92 (1.50)	3.47 (1.44)	3.44 (1.62)	3.76 (1.52)
Clearly describing the study objective (T5)	6.36 (0.94)	6.44 (0.88)	6.43 (0.86)	6.58 (0.83)	6.37 (0.82)	6.47 (0.75)	6.46 (0.83)
**Theory**	**5.71 (1.39)**	**5.71 (1.34)**	**5.59 (1.53)**	**5.65 (1.56)**	**5.35 (1.57)**	**5.43 (1.63)**	**5.53 (1.55)**
Using an appropriate study design (D1)	6.31 (0.84)	6.44 (0.95)	6.51 (0.81)	6.51 (0.93)	6.59 (0.72)	6.64 (0.62)	6.54 (0.80)
Clearly describing the sample population (D2)	5.83 (1.09)	5.44 (1.25)	5.93 (1.16)	5.98 (1.14)	5.85 (1.11)	5.96 (1.09)	5.89 (1.14)
Reporting pre-study calculation of sample size (D3)	4.47 (1.55)	4.31 (1.53)	4.42 (1.65)	4.67 (1.56)	4.10 (1.42)	4.29 (1.58)	4.38 (1.56)
Having a large sample size (D4)	4.90 (1.34)	4.57 (1.45)	4.28 (1.43)	4.33 (1.46)	4.58 (1.32)	4.42 (1.40)	4.45 (1.41)
Using a complex design (D5)	3.03 (1.27)	2.80 (1.32)	2.83 (1.63)	2.85 (1.52)	2.56 (1.45)	2.65 (1.42)	2.75 (1.48)
Pre-registering (part of) the study (D6)	4.78 (1.49)	4.31 (1.54)	3.70 (1.76)	3.70 (1.56)	3.52 (1.64)	3.77 (1.62)	3.80 (1.66)
**Design**	**4.89 (1.65)**	**4.65 (1.75)**	**4.61 (1.91)**	**4.67 (1.87)**	**4.53 (1.89)**	**4.62 (1.88)**	**4.63 (1.86)**
Achieving a high response rate (C1)	4.25 (1.32)	3.85 (1.45)	3.91 (1.48)	4.06 (1.41)	4.13 (1.32)	4.09 (1.32)	4.05 (1.38)
Achieving high statistical power (C2)	4.41 (1.43)	4.44 (1.41)	4.44 (1.53)	4.73 (1.40)	4.95 (1.31)	5.02 (1.40)	4.74 (1.43)
Applying complex statistical analyses (C3)	3.61 (1.58)	3.31 (1.33)	2.93 (1.56)	3.24 (1.66)	2.91 (1.55)	2.87 (1.51)	3.06 (1.57)
Providing open data (C4)	4.42 (1.94)	4.80 (1.76)	4.10 (1.78)	3.94 (1.81)	4.07 (1.78)	4.37 (1.67)	4.19 (1.79)
Providing open research materials (C5)	4.68 (1.91)	5.00 (1.78)	4.40 (1.67)	4.29 (1.83)	4.34 (1.67)	4.61 (1.63)	4.47 (1.73)
Providing open analysis (C6)	4.47 (1.69)	4.24 (1.87)	3.94 (1.77)	3.99 (1.73)	4.04 (1.65)	4.20 (1.59)	4.09 (1.70)
**Conduct**	**4.31 (1.68)**	**4.27 (1.70)**	**3.95 (1.71)**	**4.04 (1.70)**	**4.07 (1.67)**	**4.20 (1.66)**	**4.10 (1.69)**
Adequately describing all used stat. procedures (A1)	5.73 (1.42)	5.89 (1.24)	5.94 (1.23)	6.13 (1.07)	6.09 (1.03)	6.03 (1.03)	6.02 (1.13)
Using the appropriate statistical analyses (A2)	6.36 (1.11)	6.56 (0.77)	6.40 (0.98)	6.42 (0.98)	6.41 (0.87)	6.52 (0.71)	6.44 (0.90)
Distinguishing conf. from expl. statistical analyses (A3)	5.00 (1.69)	5.33 (1.43)	5.31 (1.31)	5.38 (1.45)	5.36 (1.49)	5.71 (1.20)	5.40 (1.41)
Reporting assumption checks (A4)	5.12 (1.27)	4.87 (1.49)	4.93 (1.40)	4.97 (1.28)	5.13 (1.25)	4.90 (1.42)	4.99 (1.34)
Clearly presenting the statistical results (A5)	6.20 (1.10)	6.31 (0.99)	6.35 (0.95)	6.30 (1.04)	6.31 (0.99)	6.46 (0.73)	6.34 (0.96)
Providing CIs for the main stat. results (A6)	5.10 (1.37)	4.91 (1.26)	4.92 (1.50)	4.94 (1.48)	4.99 (1.35)	5.07 (1.44)	4.98 (1.42)
Providing effect sizes for the main stat. results (A7)	5.29 (1.37)	5.41 (1.32)	5.51 (1.36)	5.70 (1.36)	5.60 (1.21)	5.86 (1.17)	5.62 (1.29)
Providing statistical results without errors (A8)	5.98 (1.32)	5.70 (1.35)	5.95 (1.29)	6.19 (1.15)	6.11 (1.33)	6.18 (1.07)	6.07 (1.24)
Observing large effect sizes (A9)	3.73 (1.83)	3.41 (1.30)	3.02 (1.69)	3.22 (1.86)	3.33 (1.72)	3.53 (1.82)	3.32 (1.76)
Observing main effect in hypothesized direction (A10)	3.97 (1.93)	3.93 (1.90)	3.25 (2.06)	3.35 (2.13)	3.47 (1.83)	3.49 (2.06)	3.47 (2.01)
Reaching stat. sign. of results of main hypothesis A11	4.00 (2.17)	3.09 (1.97)	2.94 (1.98)	3.03 (1.92)	3.55 (1.92)	3.46 (1.96)	3.29 (1.99)
Drawing correct conclusion from the stat. analysis (A12)	6.44 (1.15)	6.46 (1.06)	6.63 (0.65)	6.64 (0.89)	6.63 (0.64)	6.73 (0.50)	6.63 (0.76)
**Analysis & presentation**	**5.24 (1.76)**	**5.16 (1.77)**	**5.09 (1.91)**	**5.19 (1.91)**	**5.25 (1.77)**	**5.33 (1.79)**	**5.21 (1.83)**
**Total**	**5.06 (1.72)**	**4.96 (1.75)**	**4.84 (1.89)**	**4.92 (1.88)**	**4.87 (1.81)**	**4.97 (1.82)**	**4.92 (1.84)**

On average, theory ranked highest on importance, closely followed by analysis and presentation (0.2 SD lower), with even lower average scores for design (−0.5 SD) and conduct (−0.9 SD). However, as characteristics within domains varied substantially, it is more meaningful to list those that were considered more important. These were (with average scores above 6): T3 (‘explicitly stating the hypotheses to be tested’), T5 (‘clearly describing the study objective’), D1 (‘using an appropriate study design’), A1 (‘adequately describing all used statistical procedures’), A2 (‘using the appropriate statistical analyses’), A5 (‘clearly presenting the statistical results’), A8 (‘reporting the statistical results without errors’), and A12 (‘drawing the correct conclusion from the statistical analysis’). Considered less important (average scores below 4) were: T4 (‘using a large number of literature references’), D5 (‘using a complex design’), C3 (‘applying complex statistical analyses’), A9 (‘large effect sizes’), A10 (‘effect in hypothesized direction’), and A11 (‘statistical significance’).

We also listed characteristics related to the characteristics we manipulated in the vignette part of this study. For sample size, these were characteristics D4 (large sample size) and C2 (high statistical power). In all six conditions, these characteristics were rated as moderately important for quality assessment. The characteristics related to statistical significance (A9-A11; large effect sizes, effect in hypothesized direction, statistical significance) were rated as having little importance, whereas the characteristic related to reporting error (A8, ‘reporting the statistical results without errors’) was of high importance in all conditions.

Table 8 shows the correlations between the mean ratings for the conditions we compared in research questions 6–13 (see Fig 1), to establish the pair-wise association of all 29 characteristics between pairs of conditions. Correlations were high (*r* > .9), indicating strong association between mean importance ratings, which is also confirmed by visual inspection of Fig 3. Participants in all conditions seemed to agree with respect to their importance ratings of the different characteristics, regardless of manuscript type (thesis or submitted article), type of evaluation (quality or grading/publishability), and regardless of career phase (students or academics). Correlations between on the one hand thesis quality and thesis grading and on the other hand between article quality and article publishability were all very high (>.93, most > .99) and hence not distinguishable (RQ10). Within-category correlations were generally less strong for the research conduct category.

**Table 8:** Correlations Between Mean Importance Ratings Within Categories (Theory, Design, Conduct, Analyses & Presentation) and Across Categories (Total).

	RQ 6	RQ 7	RQ 8	RQ 9	RQ 11	RQ 12	RQ 13
	Academics				Students	Thesis Quality	Thesis Grading
	Thesis Quality- Grading	Article Quality- Publish.	Quality Thesis-Article	Thesis Grading -Article Publish.	Thesis Quality- Grading	Students- Academics	Students- Academics
Theory	.995***	.998***	.995***	.999***	.996***	.992***	.989**
Design	.998***	.996***	.990***	.992***	.986***	.942**	.956**
Conduct	.934*	.982***	.953**	.964**	.905	.937*	.663
Analysis & presentation	.998***	.992***	.996***	.994***	.971***	.981***	.984***
**Total**	.996***	.994***	.982***	.982***	.971***	.972***	.962***

*Note.* * indicates *p* < .01, ** indicates *p* < .005, *** indicates *p* < .001

Although correlations were very high, average importance ratings of manuscript characteristics sometimes differed across conditions, as can be seen in Fig 3 as well as Table 7. Focusing on differences of at least 0.5 standard deviation ($\frac{({SD}_{\text{1}}+{SD}_{\text{2}}}{\text{2}})$, using a large number of literature references (T4) was considered more important by students for the quality and grading of theses than by academics for the quality and publishability of articles. Interestingly, preregistration (D6) was rated by students as more important for the quality of a thesis than by academics for anything (all four conditions), and as more important by students for the grading of theses than by academics for the quality of an article.

## Discussion

Peer review is an important gatekeeper of the quality of published scientific literature. However, little is known about the manuscript characteristics reviewers consider most important when evaluating manuscript quality, whether academics differ in their peer review and their evaluation of master’s theses, and if students differ from academics in their evaluation of scientific manuscripts. This study therefore aimed at getting more insight in the manuscript characteristics that are most relevant to students and academics when they assess the quality of scientific manuscripts.

Our study showed that sample size had a small positive effect on quality assessment. In the survey study, respondents considered sample size only of moderate importance. Statistical significance did not influence quality assessment in our vignette study. Only a small number of respondents considered it a relevant aspect. In the survey study, characteristics related to the magnitude, direction, and statistical significance of the results were rated of low importance. Especially perceived lack of importance of statistical significance in quality assessment is surprising, since published research often report statistically significant findings in a context of low statistical power. The overabundance of statistically significant results in the literature suggests that statistical significance plays a larger role in manuscript submission but a smaller role in quality assessment. This is consistent with Augusteijn et al. [47], who found that statistical significance of the main result did not predict thesis grade.

We, however, should be careful generalizing the conclusions based on our results of our vignette study. An abstract that only lists the hypotheses and results might not be a sufficient proxy for an entire manuscript. Reviewers’ responses might be different to an article that builds towards a specific hypothesis and provides a nuanced discussion of the results. However, we anticipated that asking subjects to read complete research articles would result in a sample too small to obtain reliable results and thus we chose to present them only with abstracts. The lack of importance of statistical significance found in the survey may also be due to socially desirable answering since researchers might believe that statistical significance *should* not matter. And finally, at a more general level, other manipulations of statistical significance, sample size, and statistical reporting error may also lead to other results than we found in our vignette using an abstract of a scientific manuscript.

In our vignette study, the reporting error did not influence quality rating. According to the survey study, however, error-free reporting was of high importance. Perhaps participants did not spot the reporting error in the abstract, so that it did not affect the quality ratings. Schroter et al. (2008) found that reviewers have a hard time detecting errors in studies, explaining the high prevalence of reporting errors in the literature [54]. Tools such as statcheck, which automatically extract statistics from articles and recomputes *p*-values based on test statistics [55] may be valuable. Statcheck could assist the reviewers and editors in detecting such errors. Another possible solution could be to include statistical experts earlier in the research process [56,57]. Ideally already assisting when planning the research, but also in the review process. No interactions were found between the three manipulated characteristics and the text type (thesis or submitted manuscript, hypothesis 2) or career phase (student or academic, hypothesis 3).

We found that overall quality rating of manuscripts varied considerably (Table 4), confirming lack of agreement in peer reviews found in other research projects [38–40]. Participants varied in the aspects they considered influential to their quality rating and even contradicted one another. For example, some respondents considered the abstract excellently written while others considered the same writing very bad. Likewise, some considered the choice of manipulation was poorly explained but others considered the same manipulation to be very good. We did not study the possible explanation of these differences but conclude that quality assessment varies between reviewers, so that the results support the popular conjecture that the editor’s choice of reviewers can affect acceptance or rejection of a manuscript for publication.

Our survey revealed large agreement of the importance ratings *between* the respondents in the different conditions. Importance ratings of the different characteristics were similar for theses and submitted articles, students and academics, and different types of evaluation. This suggests that academics uphold similar quality criteria for master’s theses as for manuscripts submitted for publication but the bar is set lower for master’s theses. Students successfully gauged the characteristics important to their supervisors, suggesting that academics successfully passed on their own quality criteria to students. Krishna and Peter [46] showed that students’ attitude and behavior towards QRPs and RRPs is determined by the attitude they perceive from their teachers. Others found that type of mentoring influenced the probability of engaging in QRPs and RRPs [58–60]. Assessments of quality and of publishability of an article were highly similar with respect to importance of characteristics. This is surprising, since it is a common conception that there are more interests at play for publication than research quality alone [(e.g., 33, 61)].

Characteristics related to theory were considered most important in all conditions, whereas characteristics related to research conduct were rated least important. Most important individual characteristics were ‘drawing the correct conclusions’ (A12), ‘appropriate study design’ (D1), ‘describing study objectives’ (T5), and ‘using appropriate statistical analysis’ (A2). Except for describing the study objective, these characteristics are hard to judge objectively: when are conclusions warranted, when are the study design and statistical analysis appropriate? Many-labs and multi-analyst studies have shown that the same dataset and research question give rise to various ways of data analysis, rendering assessment whether data were analyzed in ‘the appropriate way’ subjective [62].

The least important characteristics were ‘complex design’ (D5), ‘complex analysis’ (C3), ‘large effect sizes’ (A9), ‘effect in hypothesized direction’ (A10), and ‘statistical significance’ (A11). The fact that appropriateness of the design and the analysis were rated most important while complexity was rated least important is a positive result. Likewise, characteristics related to the strength of the results were rated least relevant. However, the literature shows an overabundance of statistically significant results, and researchers’ use of QRPs suggests that what people believe is important is not necessarily what is reported. Atkinson et al. [45] found similar results in their experimental study. There could be a difference between the characteristics researchers believe influences them and the characteristics that really do [63], and social desirability of answering may play a role too.

### Limitations and future research

Because we were unable to sample enough students, thus resulting in a lower statistical power, all analyses, hypotheses, and conclusions including a comparison with students should be considered exploratory. We aimed at achieving a power of .80 but only achieved a power of .18 for a small effect (*f* = .10) for those tests that only include students. Our results strongly suggest that students evaluate the quality of manuscript characteristics like researchers, but larger sample sizes are recommended to confirm these exploratory results. However, we also should be careful drawing general conclusions on the similarity of the evaluation of researchers and students, as researchers and students were recruited with different methods that may have resulted in sampling bias. The academics sample enabled a power of .74 to detect a small effect, whereas the analyses including all 800 respondents had a power of .81.

The samples of academics and students may be biased, possibly due to students and academics participating who are interested above average in meta-science and concerned with good research practices. The abstract we used in the vignette may not be a valid proxy for a scientific article. Readers often use an abstract alone to assess the value of an article [27], and therefore future research might focus on the issue of whether quality assessment of an article based on an abstract alone is a good predictor of the quality of the whole manuscript.

Our survey study might have lower ecological validity than studies with an observational or experimental setup. Unlike participants in [34] and [45], our participants were aware that they were participating in a study. The study characteristics academics find important to the manuscript’s quality are not necessarily the characteristics that influence the assessment the most when peer reviewing a manuscript. For instance, complex design and complex analysis were considered least relevant for manuscript quality in our survey, but Armstrong [42] suggested that reviewers are influenced by these false cues. In our vignette study, we manipulated only three aspects and presented participants with just an abstract. An experimental setup with a manipulation of all 29 aspects we investigated in our survey does not seem feasible. Future research could investigate the impact of some of the characteristics that are considered either irrelevant (e.g., complex design and complex analysis) or highly relevant (e.g., (in)correct conclusions, (in)appropriate study design, describing study objectives, and using (in)appropriate statistical analysis). The aspects that were considered highly relevant are also aspects about which reviewers often disagree. For example, the assessment of the appropriateness of the study design and the appropriateness of the analysis seem to be subject to judgment.

To gain more insight in the characteristics that increase the publishability of a manuscript, one might investigate whether the literature is homogeneous on aspects considered highly relevant (e.g., clearly describing the study objective) and heterogeneous on aspects with little relevance (e.g., the number of references). This could provide an indication that the highly relevant characteristics are a minimum requirement, whereas characteristics with little relevance are indeed arbitrary to the publishability of an article.

Finally, it would be interesting to know how our results relate to results from other fields than psychology, and how the importance of characteristics might change over time. For example, the popularity of open science and sharing materials is increasing and might in part explain the relatively large variance in the importance rating of this characteristic amongst academics.
