# Supplementary material for: The effect of manuscript characteristics on quality assessment of scientific manuscripts in psychology
Source: PLoS One. 2026 Sep 15;21(9):e0355938. doi: 10.1371/journal.pone.0355938 (PMC13577479; doi:10.1371/journal.pone.0355938)
Supplement: Supplementary file 3 [file pone.0355938.s004.docx]

**Response to comments on our second revision of “Quality assessment of scientific manuscripts in peer review and education” (PONE-D-24-57377R2)**

*We thank the editor and the second reviewer for their comments. Below, we respond to each comment in italics, while using normal font for the comments of editor and reviewer.*

COMMENTS OF THE EDITOR

Thank you for submitting your manuscript to PLOS ONE. After careful consideration, we feel that it has merit but does not fully meet PLOS ONE’s publication criteria as it currently stands. Therefore, we invite you to submit a revised version of the manuscript that addresses the points raised during the review process.

While you have addressed most of the reviewers’ concerns, a few minor questions remain and require your attention before a final decision can be made.

*The minor questions of Reviewer 2 are good questions; we addressed them in our paper and response letter. Reviewer 3 indicates that all comments have been addressed.*

**Reviewer #2**

I found the comments from my last review to have been at least partially addressed.

I do note that I strongly disagree with the authors’ statement in the response to reviewers that different statistical errors, topics or results would be “slight changes in the manipulation” and thus unlikely to change the observed effect.

Statistical errors range from subtle to obvious, and their detectability (which is a prerequisite for taking them into account in the evaluation of an abstract) should be expected to vary accordingly. I do think that the one used in the vignette is on the subtle side, and I’d thus put much more emphasis on this limitation when discussing the results.

*We believe Reviewer 2’s comment here is based on a misunderstanding, and we are sorry for this. Our statement in the response to reviewers*

*(“We do not agree that ‘it may well be the case that’ rather slight changes in the manipulation may bring about very different results.”)*

*was a response to this comment of Reviewer 2:*

*“It may well be the case that, for different statistical errors, different p values, different results, or different abstract topics, the results found would be very different.*”

*We stated in our response letter that*

*“In our experiences with replicating studies, slight changes in the manipulation cause at most slight (and sometimes to medium) changes in effect size.”*

*We are still convinced that changes of the numbers in the abstract of the vignette are unlikely to change our results.*

*But we DO agree with Reviewer 2 that the manipulation in the vignette is on the subtle side and that other bigger manipulations may affect the results. Note that we also changed our manuscript in the first revision, as explained in our previous response letter (underlined):*

*We do actually agree with you that different manipulations may lead to different results. Hence, we changed the text in our manuscript in the following way, in the paragraph on the generalizability of our results (starting at the last sentence of p.38):*

*“And finally, at a more general level, other manipulations of statistical significance, sample size, and statistical reporting error may also lead to other results than we found in our vignette using an abstract of a scientific manuscript.”*

But I think that this will be clear to the reader from the description of methods and results, so I won’t be fighting the authors further on this issue.

*No, please, no fighting, we agree with you.*

That said, I have found a problem which I had overlooked in previous reviews, which I think is quite important and does have to be fixed before publication. Although the study was entirely conducted with psychology students and researchers, the fact that they come from this research field is nowhere to be found either in the title or abstract, which only mention “students” and “academics” with no information on their background. I’d thus strongly advise the editor and authors to mention this both in the title (e.g. “The effect of manuscript characteristics on quality assessment of scientific manuscripts in psychology”) and abstract (e.g. “…psychology academics give of articles submitted for publication, and psychology academics and students…”).

*Reviewer 2 is completely right. Thanks. We changed the title and the abstract as suggested by Reviewer 2. We also made it explicit that the theses are written by psychology students. The first sentence of the abstract now reads:*

*“We report a vignette study and a survey to investigate which study characteristics influence quality ratings psychology academics give of articles submitted for publication, and psychology academics and students to theses written by psychology students.”*
