# Supplementary material for: The effect of manuscript characteristics on quality assessment of scientific manuscripts in psychology
Source: PLoS One. 2026 Sep 15;21(9):e0355938. doi: 10.1371/journal.pone.0355938 (PMC13577479; doi:10.1371/journal.pone.0355938)
Supplement: Supplementary file 4 [file pone.0355938.s002.docx]

**Response to comments on “Quality assessment of scientific manuscripts in peer review and education” (PONE-D-24-57377)**

*We thank the editor and reviewers for their comments. Below, we respond to each comment in italics, while using normal font for the comments of editor and reviewers.*

COMMENTS OF THE EDITOR

Thank you for submitting your manuscript to PLOS ONE. After careful consideration, we feel that it has merit but does not fully meet PLOS ONE’s publication criteria as it currently stands. Therefore, we invite you to submit a revised version of the manuscript that addresses the points raised during the review process.

In your review, please provide detailed answers, particularly regarding the statistical analyses and the application of scale, domains, and scores, as discussed by both reviewers. Among Reviewer #2’s concerns is the use of frequentist analysis for most of the study without reasonable explanation. Please explain the reasoning behind choosing this statistical method for your hypotheses.

*We explain this in our preregistration and in the ‘statistical analysis’ section of our paper: “Since hypotheses 1c, 2c and 3c indicated no effect and null hypothesis significance testing cannot provide support for null hypotheses, we evaluated these hypotheses using Bayesian statistics.” (p23, line 3).*

Both reviewers have also expressed skepticism about sample validation, recruitment, and data interpretation. Reviewer #1 is also concerned about how readers should interpret your scores and criteria to assess the quality of abstracts, manuscripts, and theses, as they do not necessarily reflect peer review practices.

*We address all these points below.*

2. We note that the grant information you provided in the ‘Funding Information’ and ‘Financial Disclosure’ sections do not match.

*We aligned both sections, stating the same information.*

 3. Thank you for stating the following financial disclosure:  Research Talent Grant of the Dutch Science Foundation (NWO). Please state what role the funders took in the study.  If the funders had no role, please state: "The funders had no role in study design, data collection and analysis, decision to publish, or preparation of the manuscript."

*We added this sentence and added it to our cover letter.*

4. Please note that your Data Availability Statement is currently missing the repository name and/or the DOI/accession number of each dataset. If your manuscript is accepted for publication, you will be asked to provide these details on a very short timeline. We therefore suggest that you provide this information now, though we will not hold up the peer review process if you are unable.

*Both the data and the codebook of the data can be found at osf.io/j2gpk, as indicated in our manuscript.*

 5. Your ethics statement should only appear in the Methods section of your manuscript. If your ethics statement is written in any section besides the Methods, please move it to the Methods section and delete it from any other section. Please ensure that your ethics statement is included in your manuscript, as the ethics statement entered into the online submission form will not be published alongside your manuscript.

*We entered the ethics statement in the Methods section (i.e., in the subsection “Ethical review and data management”). (p17)*

 6. We note that you have referenced “Cooper H, DeNeve K, Charlton K.”  which has currently not yet been accepted for publication. Please remove this from your References and amend this to state in the body of your manuscript: (ie “Cooper H, DeNeve K, Charlton K.” [Submitted]”) as detailed online in our guide for authors

*This is a mistake, this paper has been published in 1997, and is referenced accordingly in our paper:*

*“Cooper, H., DeNeve, K., & Charlton, K. (1997). Finding the missing science: The fate of studies submitted for review by a human subjects committee. Psychological Methods, 2(4), 447.”*

7. Please include captions for your Supporting Information files at the end of your manuscript, and update any in-text citations to match accordingly. Please see our Supporting Information guidelines for more information: <http://journals.plos.org/plosone/s/supporting-information>.

*Thanks, but we do not have any Supporting Information files.*
 
Reviewers' comments:
Reviewer's Responses to Questions

**Comments to the Author**
1. Is the manuscript technically sound, and do the data support the conclusions?

Reviewer #1: No

Reviewer #2: Partly

*We adapted our conclusions, for instance, emphasizing that reviewers only evaluated an abstract and not a full article.*

2. Has the statistical analysis been performed appropriately and rigorously?

Reviewer #1: No

Reviewer #2: No

*We explained the logic of our statistical analyses and made clear that all were preregistered in detail. We also explicitly link to the preregistration in our paper.*

3. Have the authors made all data underlying the findings in their manuscript fully available?

Reviewer #1: Yes

Reviewer #2: Yes

4. Is the manuscript presented in an intelligible fashion and written in standard English?

Reviewer #1: Yes

Reviewer #2: Yes

**Reviewer #1 (R1):** Thank you for the opportunity to review this manuscript. The following are my recommendations for its improvement:

Title
1. As this is not about evaluation of actual manuscripts, I would suggest changing the title to: Exploring students and researchers rating of the quality of manuscripts

*R1’s comment made us rethink the title of our paper. If we must summarize our paper in one sentence, it would be “the effect of three manuscript characteristics (error, statistical significance, sample size) reported in abstracts on manuscripts’ rated quality (part 1), and the effect of manuscript characteristics on scientific manuscripts’ rated quality (part 2)”. Ideally, the title reflects this sentence.*

*R1 is right that no scientific manuscript is evaluated in part 2, whereas in part 1 the quality of abstract of a scientific manuscript is evaluated. Note that the title proposed by R1 misses the essential “characteristics of…” that is essential to both parts of our study. This also holds for our original title. To improve upon both the original title (“Quality assessment of scientific manuscripts in peer review and education”) and the title proposed by R1, we changed the title to:*

*“**The effect of manuscript characteristics on quality assessment of scientific manuscripts”*

*Note that we skipped the “in peer review and education” as it is not essential.*

*We believe that the fact that no complete manuscripts are evaluated by respondents is clearly explained in the abstract, as the abstract describes that abstracts are evaluated (part 1) and “the importance of 29 manuscript characteristics … for assessing either the quality of a manuscript or its publishability (article) or grade (thesis).” (part 2). Moreover, the R1’s proposed title also does not make clear that no complete manuscripts are assessed.*

*Also, in the main text we added more explicitly that respondents evaluated the abstract of a manuscript, for instance “In our vignette study, we focused on three research characteristics that we manipulated in a study’s abstract to study examine their effect on the quality evaluation of scientific manuscripts (article or thesis).” (p9, line 15, added text underlined)*

*Finally, we added as a last sentence to the abstract: “Further research is needed to examine which study characteristics researchers consider most important when assessing a manuscript’s quality in peer review.” (p2, line 16)*

Abstract
1. Please include information on the quality scores given to vignettes, and quantify the influence of the sample size you mention. Additionally, specify if these were actual existing abstracts/manuscripts, or created for this experiment

*As requested, we added information on the quality scores in the abstract:*

*“In the vignette study, 800 respondents evaluated the quality of an abstract (7-point scale) of studies ...”. (p2, line 4)*

*And we quantified the sample sizes by adding them to the sentence between brackets:*

*“… quality of an abstract (7-point scale) of studies with small (N = 54) or large sample sizes (N = 540)”. (p2, line 5)*

*We do not need to explicitly specify whether the abstracts were existing or created, as our use of a vignette study and the manipulation of three manuscript characteristics already implies that we created the abstracts.*

2. “These results suggest that researchers’ assessments of manuscript quality are not responsible for publication bias.” – I disagree this study was not designed to assess publication bias.

*In retrospect, we are also not content with the formulation of our cited sentence, as it is too general and does not directly follow our results. We deleted the sentence from the abstract.*

3. “are already adopted” – I find this conclusion wrong, this might indicate that science educations implies certain standardized quality considerations. I do not find adopted as a proper word in this context. We know from rant review, that when we specify domains and provide concrete ratings, researcher can apply those, same as teachers in grading students or their coursework. Why would researchers need to adopt anything, when they are not ask to rate manuscripts in this way in everyday research publishing or review.

*We do not understand R1’s comment here. We believe the sentence does not imply that researchers have a standard(ized) list of criteria, but that students and teachers seem to apply criteria similarly.*

*We state in the abstract that “quality criteria … are already adapted by students”, but R1 talks here about researchers. As our paper shows, students and researchers use highly similar ratings of the importance of different aspects relevant to quality assessment of articles and theses. We therefore believe that our suggestion (and not a conclusion) that students adopt similar criteria (i.e., similar to the criteria of teachers) is sensible. As we think ‘adopt’ is preferable to ‘apply’, we decided not to follow upon R1’s suggestion this time.*

Introduction
1. I believe authors have to discuss the influence of registered reports on the reporting of non-significant results, and current data on this, similarly they can comment on the results of clinical trials that have to be registered by law – i.e. researchers may chose to pursue studies for which their pilot analysis already indicate potential positive effect.

*We thank R1 for the suggestion but did not include it in our manuscript as we do not see its direct relevance for our paper on “The effect of manuscript characteristics on quality assessment of scientific manuscripts”. Moreover, because R2 found the introduction long, and rather liked it, we did not expand it.*

Peer Review
1. There are approximately 33,100 peer-reviewed – Please provide citation for this, current estimates are around 70,000 using Open Alex data. Similarly we have more than 6 million articles published per year, using OpenAlex or Dimensions

*The number of active academic peer-reviewed English-language journals has been growing and will likely continue growing. We therefore believe the added value of numbers like this are limited. We therefore deleted the three sentences from our manuscript on how many journals there are, and articles per year, and what is the average acceptance rate of submitted manuscripts in psychology.*

2. As the authors stated “we focus on the question which manuscript characteristics are most important for manuscript quality rating that might differ between reviewers and even between manuscripts.” – In regards to this I would greatly revise the current introduction and peer review section, and provide literature review on published papers that aimed to assess manuscript or peer review quality, and report on methods used to do so, and domains assessed in previous research.

*Our manuscript already provides a discussion of the literature (see references 27-43), focusing on conclusions that we believe are relevant for our manuscript. And we do not want to expand the introduction, also as R2 found it rather long.*

3. “also tested what influenced their quality assessment” – was this testing part of the original study when the original question was asked? If not, this should be clearly stated.

*We changed the sentence to (added text is underlined):*

*“We did not only investigate the beliefs of academics and students, but also tested what influenced their quality assessment in the vignette study that we preregistered together with the survey study*.” (p9, line 12)

Participants
1. Please report what was the original plan for not meeting the sample size. You stated you needed at least 787 participants, and you obtained 687 – why did you not conduct another or several more rounds of invites?

*Page 12 of the preregistration explains the power analysis in more detail. Following our preregistration we now added the sentence “Assuming 33 participants per condition, this meant 528 academics and 264 students.” (p13, line 1)*

*Note that our 687 academics exceed the required 528 academics. Our ‘Participants’ section explains that we achieved this number in our third wave of data collection.*

*Our ‘Participants’ section also explains in detail how we attempted to collect enough student data in a period of 11 months (!), and that (because of the low number of responses and the subsequent low statistical power to detect small effect sizes) we consider the analyses that include students as exploratory rather than confirmatory.*

2. “ even when their main field of research was not within a research field in psychology” – Please spicy if this was originally planed like this, or were you aiming to collect responses only from individuals with primary field of psychology.

*Thanks for noting this. We deviated from our preregistration (exclusion criteria on page 12) and did not appropriately specify this in our paper. Hence, we changed our manuscript:*

*“Many researchers (256, 37.2%) indicated they did not work in any of the pre-specified research areas, even though all participants had published on topics within the pre-specified area of psychology. Deviating from our preregistration, we decided not to remove participants from other fields, thereby retaining the planned statistical power for our analyses.” (p15, line 3)*

3. Due to later statement “in which psychology research field they primarily worked”, I would appreciate a sub-analysis of results, with those not primarily working in psychology being excluded, and compared to results of those that do.

*We also ran all statistical analyses without the participants that did not primarily work in psychology, that is, we tested all hypotheses H1a-c to H3a-c both with and without these participants. An excel file that includes both the results of the analyses with and without these participants can be found at* <https://osf.io/84ugb>*. The substantive conclusions are the same for all 3×3 hypotheses tests. We added to the text of our paper:*

*“Deviating from our preregistration, we decided not to remove participants from other fields, thereby retaining the planned statistical power for our analyses (note: tests of hypotheses H1a-c to H3a-c excluding those participants yielded the same substantive conclusions, see* [*https://osf.io/84ugb)*](https://osf.io/84ugb))*.” (p15, line 5)*

4. Table 1, I would suggest that the reporting is changesdto N (%) with 0 decimal points, or on the reverse order, the use of n=, seems unnecessary.

*We changed to N (%), removed the “n=”, but kept one decimal point for the percentages.*

5. Table 3 – I dislike the phrasing - Using a large number of literature references., I would find this to have a QRP associated with it, Using relevant lit. references seems more important than the number.

*We asked participants about manuscript characteristic “using a large number of literature references” and cannot change that. We understand R1’s comment about ‘relevance’ in principle being more important than the ‘number’ of literature references.*

Similarly phrasing of using complex design and using complex analyses, seem out of place in the list. The researchers should be aware of the pyramid of evidence, which of those do the drafters consider to the complex? Why Is using complex analysis in C, when using appropriate analysis is in A – this seems to be bad design of the scale and domains. What reliability testing has been done to confirm that these items belong under the same domains?

*The 29 components as well as their categorization were discussed by the authors as well as our meta-research group (*[*https://metaresearch.nl/*](https://metaresearch.nl/)*). We also used our experiences in related projects, see, for instance, the two following papers:*

*Wicherts JM, Veldkamp CL, Augusteijn HE, Bakker M, Van Aert R, Van Assen MA. Degrees of freedom in planning, running, analyzing, and reporting psychological studies: A checklist to avoid p-hacking. Frontiers in psychology. 2016;7,1832. Available from:* [*https://doi.org/10.3389/fpsyg.2016.01832*](https://doi.org/10.3389/fpsyg.2016.01832)*. (cited 911 times, google scholar, May 1 2025)*

*van den Akker, O. R., Bakker, M., van Assen, M. A., Pennington, C. R., Verweij, L., Elsherif, M. M., ... & Wicherts, J. M. (2024). The potential of preregistration in psychology: Assessing preregistration producibility and preregistration-study consistency. Psychological Methods.*

*But it may indeed be the case that we missed some possible manuscript characteristics that may also be important, or that some researchers believe that the characteristics can be categorized differently. That is why we also conducted (and preregistered, see page 15 of the preregistration) a PCA.*

It seems evident from the way respondents answered the question on what influences the ratings, that they might not adhere to the domains your teams have devised. In a registered report or protocol with statistical analysis plan predefined, is using the appropriate statistics part of design or only of analysis? Why is providing code or data part of conduct and not of presentation? Why is describing the sample part of design and not presentation? You had a planned sample, and you did not reach it. The sentence “Clearly describing the sample population” – can only occur after analysis of the results, so it can not be part of design as Table 3 would imply.

*The categorization of manuscript characteristics will always be subjective to a certain extent. But this is less relevant for our main analyses, because we analyzed the data at the item-level for each condition separately (as the 29 manuscript characteristics did not sufficiently correlate).*

In short, please indicate in the survey itself, were respondents presented the sentences with the domains or only sentences?

*The manuscript characteristics were presented to participants in a random order, that is, not clustered by domain. This is also mentioned in our manuscript (“All 29 characteristics were presented in a random order different for different participants.” (p21, line 5))*

I was not surprised to read later that the KMO scores were low, as these domains to me do not meet face validity.

*The low KMO scores have nothing to do with how we characterized the domains, but they are the result of the relatively low correlations between participants’ answers to the 29 manuscript characteristics. That is, whatever our characterization of the domains, the KMO scores remain the same.*

6. Since hypotheses 1c, 2c and 3c indicated no effect, we evaluated these hypotheses using Bayesian statistics. – Please indicate if this analysis was preregistered? If not, this should be moved to appendix.

*Yes, these are preregistered, see page 17 of our preregistration. The preregistration also includes the code of our analyses. At the start of our “statistical analysis” section in our paper we state that our analyses (including the code) are preregistered.*

7. I would suggest putting content coding as a subtitle in the analysis section

*As the content coding refers to only a minor part of our analyses, and only a few lines of the statistical analysis section, we did not use a subtitle for this part. Note that we also do not use subtitles for the other parts of our statistical analysis.*

Results
1. Please indicate in the stat. section if the quality rating data was normally distributed to report M and SD, instead of Md and IQR?

*It does not matter if the quality ratings are normally distributed. The sampling distribution of the mean will be normally distributed, by the central limit theorem, as the sample size is large. Even for population distributions that deviate a lot from the normal distribution, the approximation of the sampling distribution of the mean by the normal distribution are close for N > 100. Our sample size far exceeds 100.*

*Similarly, the sampling distribution of regression coefficients is well approximated by a normal distribution even though the distribution of the error is not normal, again by the central limit theorem.*

*Finally, our main analyses use the interval scale properties of the data when computing correlations and conducting regression analyses. On the other hand, median and IQR use the ordinal scale properties and are therefore not in line with our other analyses.*

*Hence, we see no reason to report Md and IQR, or statistics related to the (approximation of the frequency distribution by the) normal distribution.*

2. Reporting of aspects – would it be possible for you to showcase the first choice of the most important aspect of participants, then the second, then the third?

*Your comment concerns Table 6. We do not have these data, that is, we asked participants to rate the three most important aspects but not to rank them among each other.*

I am not sure that the coding you applied matches the domain coding of researchers themselves. You separated Reporting of statistical results, statistical significance, and statistical reporting of error, from results reporting. And yet in Table 3, these items fall under domains of conduct or analysis and presentation. I would have expected your own top domains to be identical. You state “other (e.g. preregistration, open data)”, but in Table 3 this is part of conduct, which I disagree with conceptually, to me this is part of presentation, but at least you should apply your own consistent coding and justification behind it.

*You are right that there is no clear or straightforward link between the categories in Table 3 and Table 6. But such a link is impossible, as researchers’ responses often cannot be directly linked to the categories used in Table 3. For instance, ‘writing’ (second in Table 6) does not occur in Table 3. As directly linking was not possible, we used categories in Table 6 that are at a higher level than those in Table 3, such as ‘writing’. In this way it becomes clear what researchers think they find most important when evaluating the quality of manuscripts (apparently, issues related to methodology and research design, followed by writing).*

3. Results style – As you had 13 research questions, please rewrite the results in the order of those questions, with appropriate subtitles and report them in that order. I would highly recommend, that you start with, results for our 13 predefined research questions are provided in Table X or Box X, which would list each question, the answer to it, as well as strength of evidence rating for it, with indication or planned vs additional analysis to explore/confirm the RQ.

*Thank you for your comment; we realized that we did not explicitly refer back to our research questions when describing the methods and results sections. We also wrote the results in the order of the research questions, and we made that clear in the text by explicitly referring to these research questions. More specifically:*

- *All research questions are listed in the text and in Figure 1 at the end of the introduction section, with hypotheses 1-3 linked to research questions 1-3.*
- *The ‘statistical analysis’ section explains how the research questions are addressed. We added for RQ 1-3:*

*“Research questions (RQ) 1-3 were answered using linear regression analyses, by testing the main effects of manuscript characteristics (sample size (H1a), statistical significance of the effect (H1b), reporting error (H1c)), by adding to the model and testing the three interactions manuscript characteristic × manuscript type (thesis vs. article) (H2a-H2c), and by adding and testing the three interactions manuscript characteristic × actor (student vs. academic, H3a-H3c) (see preregistration for the equations).” (p22, line 6)*

- *The ‘statistical analysis’ section also explains how the other research questions are addressed:*

*“The investigation of research questions 4 to 13 was exploratory and was not part of the preregistration. To investigate these research questions, we provide descriptive statistics and correlations between the importance ratings of either the new principal components (RQ 4-5), or their four intended categories (Table 3), of the conditions compared in hypotheses research questions 6 through 13 (see also Figure 1).” (p24, line 10)*

- *The results section explicitly refers to all research questions. After the tests of the hypotheses, we state at the start the “survey section” with “… we analyzed the data at the item-level for each condition separately to answer research questions 4 and 5 (see Table 7 and Figure 3).” And later on we provide the results on RQ 6 – RQ 13:*

*“Table 8 shows the correlations between the mean ratings for the conditions we compared in research questions 6 to 13 (see Figure 1), to establish the pair-wise association of all 29 characteristics between pairs of conditions.” (p37, line 6)*

*The “discussion section” discusses our findings more generally and does not mention each of the research questions individually.*

4. “As the items in the survey did not correlate sufficiently” – Please start this section first with a little explanation. To test X, we conducted KMO analysis which indicated that the prespecified domains were no….

*The “statistical analysis” section in our paper already explains why we do this:*

*“To analyze the importance of the 29 manuscript characteristics in the survey, a PCA was planned for each of the six survey conditions separately to reduce the dimensionality of the dataset if (i) the Kaiser–Meyer–Olkin (KMO) test indicated sufficiently correlated items (KMO > .6 in all six conditions) and at the same time (ii) items loaded similarly on the principal components across the six conditions. If either of these two criteria were not met, analyses would take place at the item-level and the four intended categories (theory, design, conduct and analysis, and presentation). More details can be found in the preregistration.” (p24, line 3)*

*As we explain our analyses in the ‘statistical analysis’ section, and do not want to repeat ourselves, we did not explain again why and how we use the KMO index.*

As preplaned, we then analysed data at the item level. “the average rating of each category “ – why is this shown, when you have evidence that they do not belong to the same category?

*We continue using the four domains as the characteristics within each domain can be grouped based on their content. We also preregistered doing analyses at the level of domains (p15):*

*“If the PCA shows that items 7 till 35 cannot be reduced, and therefore all need to be analyzed separately, we will also analyze them per a priori intended category.” (*

*Note that we indeed analyzed all items separately and per domain. We want to emphasize that the results of Table 8 show the usefulness of analyzing the data at the domain level, showing that students and researchers agree with respect to their importance ratings of the different characteristics or categories, within and between categories, and even regardless of manuscript type (thesis or submitted article), and type of evaluation (quality or grading/publishability).*

5. “Theory was rated to be the most important” – I am not sure that this is the proper way to interpret these results. As you decided on item level presentation, list the items with most. One of the design items had a very high score too, and readers should know which of the individual items statistically differ in scores from which items? In regards to the vignette, where answers indicated that design is by far the most important aspect (when asked to list 3 aspects), we as readers should know if this second type of rating had stat. sign difference between which individual level items.

*We agree with the reviewer that a focus on domains is less appropriate because the characteristics within a domain vary considerably with respect to rated importance. Hence, we rewrote the paragraph such that we mention the average importance by domain but focus on the importance of the characteristics.*

*We do not believe in the usefulness of conducting and reporting the 29×28/2 = 406 pairwise comparisons, because it provides a lot of detailed information that we are not primarily interested in. Instead, we mentioned the characteristics that scored high (>6) and those who scored low on importance (<4), as these are very clearly separated. Our new paragraph (see lines xx-yy) is:*

*“On average, theory ranked highest on importance, closely followed by analysis and presentation (0.2 SD lower), with even lower average scores for design (-0.5 SD) and conduct (-0.9 SD). However, as characteristics within domains varied substantially, it is more meaningful to list those that were considered more important. These were (with average scores above 6): T3 (‘explicitly stating the hypotheses to be tested’), T5 (‘clearly describing the study objective’), D1 (‘using an appropriate study design’), A1 (‘adequately describing all used statistical procedures’), A2 (‘using the appropriate statistical analyses’), A5 (‘clearly presenting the statistical results’), A8 (‘reporting the statistical results without errors’), and A12 (‘drawing the correct conclusion from the statistical analysis’). Considered less important (average scores below 4) were: T4 (‘using a large number of literature references’), D5 (‘using a complex design’), C3 (‘applying complex statistical analyses’), A9 (large effect sizes), A10 (effect in hypothesized direction), and A11 (statistical significance).” (p33, line 10)*

6. Table 7 – average domain level scores should be deleted, due to KMO scoring indicating these are not the same domain. Those that had appropriate scores, can be considered for average score, if you feel that appropriate.

*We did not follow up on the reviewer’s suggestion and decided to stick to our preregistration. However, note that in our revision we focus more on the characteristics rather than the domains, compared to our first submission, as requested by R1.*

Discussion
1. I will refrain from detailed comments on discussion until the above are clarified. I would however advise authors to make it very clear that researchers are not normally asked to rate the quality of abstract/manuscripts/thesis using the scale presented in this domain. And the peer review of the manuscripts does not work in regards of improving the initial given score, to then a higher score, as many aspects of the design can rarely be changed. So I would suggest emphasizing in the introduction and discussion, that in this exercise of trying to score manuscripts, what was found.

*Yes, we are now clear on what our study does – assess the quality of an abstract in a vignette that varies three characteristics and ask in a survey what characteristics are most important for the quality of a manuscript (paper or thesis).*

Also, we have no test-rest scores for this, and we are not sure, that the same researchers would give the same score or not change their criteria when asked to do these kind of tasks regularly.

*Indeed, we do not know this.*

In hopes the above can help improve the readability and presentation of the results.

*Thanks for your comments!*

**Reviewer #2:**

The manuscript describes an interesting empirical attempt at studying some aspects of how reviewers evaluate articles or theses. Unfortunately, there are some important limitations that are not adequately addressed by the authors. I also have some issues concerning the statistical analyses for some of the hypotheses under study.

Major concerns:
1. As far as I could understand, the entire vignette study was based on a single abstract, with three elements (sample size, statistical result and reporting error) that varied between two different categories.
This poses an important restriction on any generalizations that can be drawn from the study, as one cannot generalize from this specific case how much judgments of quality by researchers or students are affected by these three factors in general.

*We agree with R2 that we should be careful with generalizing the conclusions of our paper.*

For instance, this is only one example of a reporting error (which may not be particular evident for most people): other reporting errors may be more easily noticed and have a much larger impact on quality judgments. Similarly, studies with different designs or topics may have their quality assessments more or less affected by sample size or statistical results.
Generalizing from the experimental study to general statements concerning what researchers take into account when evaluating manuscript would likely require multiple examples, which would ideally be entered into a statistical model as random variables (for more on this issue, see Yarkoni, 2020 - <https://doi.org/10.1017/s0140525x20001685>).

*Again, we agree with R2.*

This seems to me the most important limitation on what can be concluded from the study – however, it does not seem to be mentioned at all in the limitations section. The impossibility of generalizing from this single abstract to manuscripts/theses in general should be clearly stated both in the introduction and discussion, and taken into account when presenting the results.

*We now write in the discussion section (complete fourth paragraph):*

*“We, however, should be careful with generalizing the* *conclusions based on our results of our vignette study. An abstract that only lists the hypotheses and results might not be a sufficient proxy for an entire manuscript. Reviewers’ responses might be different to an article that builds towards a specific hypothesis and provides a nuanced discussion of the results. The lack of importance of statistical significance found in the survey may also be due to socially desirable answering since researchers might believe that statistical significance should not matter.” (p39, line 8)*

*We also added as a last sentence to the abstract: “Further research is needed to examine which study characteristics researchers consider most important when assessing a manuscript’s quality in peer review.” (p2, line 16)*

This is particularly important for the statistical reporting error variable, the detection of which seems to require that participants would be able to spontaneously notice that a p value does not correspond to a given t value (a correspondence which most researchers are not used to doing without the use of software or tables). I would thus strongly avoid making any kind of generalization about how much researchers perceive or value reporting errors based on this example alone.

*The literature is clear on this - researchers generally do not and cannot detect errors in statistical reporting. We discuss this in the fifth paragraph of our discussion, citing the research of Schroter et al. (2008) and of Nuijten and coworkers. Note that the results of our vignette are in line with these findings, as our participants also did not detect the reporting error, as we also discuss. Fortunately, tools such as statcheck have been developed to assist researchers in spotting reporting errors.*

2. The recruitment of researchers and students is done through very different methods: thus, any statistical comparisons may be reflecting differences in the populations induced by the recruitment methods, rather than the different career stages. Particularly, the fact that students were recruited through a method that was much more dependent on the personal contacts of the researchers raises concern that this may have selected a population that was more familiar with discussions on open science/reproducibility than the general population of researchers recruited through the Web of Science.
This should also be explicitly mentioned in the discussion section and in any comparisons drawn between researchers and students throughout the manuscript.

*Thank you for noting this. We now write in the first paragraph of our “limitations and further research” paragraph (we added the second sentence, starting with ‘However,…”):*

*“Our results strongly suggest that students evaluate the quality of manuscript characteristics like researchers, but larger sample sizes are recommended to confirm these exploratory results. However, we also should be careful with drawing general conclusions on the similarity of the evaluation of researchers and students, as researchers and students were recruited with different methods that may have resulted in sampling bias.” (p42, line 4)*

*We also want to mention here that researchers and students could not have been recruited with the same method, as students could not be recruited through papers.*

3. Research questions 6 to 13 seem not to have been addressed by the statistical analysis at all. Although the formulation of these questions is rather haphazard in the manuscript (which makes it difficult to understand some of them), the authors state that they will “study differences in the relevance of the different characteristics” when assessing quality vs. publishability, theses vs. articles, etc.

*The introduction lists all research questions.*

However, the only statistical analyses presented for these findings are correlations between the answers in the different conditions (academics vs. students, articles vs. theses, quality vs. grading/publishability). These do not test for the presence of differences between these conditions at all, as the presented p values for correlations test the (highly unlikely) null hypothesis that there would be no correlation at all between answers in both conditions, and don’t answer the intended question of whether there are differences in the assessment of different features between the conditions at the population level.

*We would like to refer to our detailed preregistration of our study (30 pages, including all research questions, hypotheses, statistical analyses, including the code to carry out our analyses). As you can read in our preregistration and in manuscript as well, we had research questions (4-13) for the survey but no statistical hypotheses.*

*We chose to examine “difference in assessment quality” by computing correlations. If the correlation of the importance ratings of characteristics A and B is very high, then we conclude that importance ratings of A and B are similar, as the order of rated characteristics is similar. As almost all our correlations in Table 8 are extremely high (>.9), we conclude that all importance ratings are similar, answering research questions 6-13.*

*We added the results of the statistical tests of the correlation (i.e., p-values) because the number of data points is small (e.g., only five for domain “theory”), and because this is regular practice. Note, however, that it is not the statistical significance what matters here, but the very large correlations between the importance ratings signaling strong agreement between academics and students when assessing what study characteristics are relevant for the quality of theses and articles.*

To answer these questions, a direct statistical comparison between the rating of different items in different conditions would be needed, in order to test the null hypothesis that there is no association between conditions and survey answers.

*The correlation is the most common statistical measure of association.*

If the authors feel that the exploratory nature of the questions and the large number of items/conditions do not warrant formal significance testing, this is fine. However, it would still be useful to present p values or confidence intervals for differences.

*We do not present confidence intervals on differences between importance ratings for two reasons. First, we are not interested in, for instance, if researchers give exactly the same importance rating in absolute numbers for “D1, using an appropriate study design” for a master thesis, as students do. We want to know if they find the same study characteristics important, and this is established by correlating their importance ratings.*

*By using Pearson’s rather than Spearman’s correlation, we also consider the relative information in the two rankings. Second, using correlations is also a much more efficient statistical analysis. Presenting confidence intervals for differences would require 8 (research questions) × 29 (items) = 232 differences with confidence intervals for information we are not mainly interested in and for which we did not formulate hypotheses.*

Moreover (and perhaps more importantly), statements such as “This suggests that academics uphold the same quality criteria for master’s theses as for manuscripts submitted for publication” are clearly not warranted based on a correlation analysis alone.

*As the correlations were very high (> .9), we maintain our position that they generally uphold the same quality criteria. The very high correlations show that the very same criteria are deemed most relevant for students and academics when assessing the quality of theses and articles.*

4. I find it quite strange to use traditional frequentist analyses for most of the article, but a Bayesian analysis for hypothesis 1c/2c/3c. If I understood correctly, this was done because the authors’ hypothesis was that there would be no effect, and thus they wanted to provide evidence in favor of the null hypothesis.
Doing this through Bayesian analyses, however, eliminates the commensurability between the analyses used for different hypotheses, as the conclusions that can be drawn from Bayesian vs. frequentist approaches are fundamentally different. The paper would thus be more coherent if either Bayesian or frequentist analyses (or both) were used for all hypotheses.

*See also our preregistration, where we explain and motivate our statistical analyses in detail. Indeed, as preregistered, we chose to test hypotheses 1c/2c/3c with the Bayes Factor, as only with Bayesian analyses can one obtain evidence in favor of the H0. We now added to our “statistical analysis” section:*

*“Since hypotheses 1c, 2c and 3c indicated no effect and null hypothesis significance testing cannot provide support for null hypotheses, we evaluated these hypotheses using Bayesian statistics.” (p23, line 3)*

Arguing in favor of the lack of a large effect shouldn’t be an obstacle to use frequentist analysis: if the authors want to evaluate evidence against an effect, they can use traditional equivalence testing for this to remain within the framework used in the rest of the article. Conversely, there is no problem in using Bayesian analysis to test directional hypotheses such as 1a/b, 2a/b and 3a/b.

*We chose to conduct traditional frequentist statistical analyses as these are still much more common in applied research. Similarly, as the Bayes Factor is more mainstream than equivalence testing, we chose the Bayes Factor. We also see no advantages of using equivalence testing over the BF – see also*

*Linde, M., Tendeiro, J. N., Selker, R., Wagenmakers, E. J., & van Ravenzwaaij, D. (2023). Decisions about equivalence: A comparison of TOST, HDI-ROPE, and the Bayes factor. Psychological Methods, 28(3), 740.*

*As we preregistered our analyses, and R2 (nor the editor nor R1) raises no fundamental issues with our analyses (i.e., we made no error), we decided to stick to our original analyses plan.*

Minor concerns:
General:
1. Figure legends seem to be missing from the manuscript file altogether (or at least I could not find them).

*Oops, what an unlucky oversight! Thanks for alerting us. We added the figure legends when resubmitting our revised paper. Note that these figure legends are in the manuscript, not in the figures.*

2. For some reason, line numbers disappear after page 29.

*Yes, strange, we noticed it too. Thanks again. We chose line numbering per page, as continuous line numbering did not work for our paper (i.e., it stopped halfway and we could not fix that).*

Introduction:
1. The introduction is very long, with a lot of general sections about peer review that frequently touches on issues that are general knowledge for most scientists and are sometimes not directly related to the research questions.
I personally found this literature review quite interesting and thorough, but also somewhat superfluous. This is a matter of style and the authors do not need to change it, but I would consider shortening the introduction to make the paper more readable.

*We are happy to read that you found the literature review interesting and thorough.*

*After briefly describing some current problems in the empirical sciences (QRPs and publication bias), we provide a literature review on peer review and review in education. We believe it is not overly long (6 pages, double spaced) and decided to keep it as it is.*

Study hypotheses:
1. The exploratory research questions (RQ4-RQ13) are formulated haphazardly and are hard to understand clearly either from the text or from Figure 1, which is hardly self-explanatory. Please consider formulating them one by one, as was done for the first research questions. Also note that RQ4 and 5 are not mentioned in the figure.

*All research questions are listed in the introduction. RQ 4 and RQ 5 are general hypotheses, whereas RQ 6 – RQ 13 are on comparing conditions, as indicated by the arrows in Figure 1. This is now also explained in the caption of Figure 1.*

*But after reading your and R1s comments we realized that we did not explicitly refer to our research questions when describing the methods and results sections. We did that now, see also our detailed response to comment 4 of R1.*

2. The correspondence between statistical analyses and hypotheses is also not clear. It seems to me that RQ2 and RQ3 are being tested through the interaction terms in the regression models, for example but it would be useful to state this clearly in the hypothesis or methods section.

*The “statistical analysis subsection” already stated “Research questions (RQ) 1-3 were answered using linear regression analyses.” We expanded this text to:*

*“Research questions (RQ) 1-3 were answered using linear regression analyses, by testing the main effects of manuscript characteristics (sample size (H1a), statistical significance of the effect (H1b), reporting error (H1c)), by adding to the model and testing the three interactions manuscript characteristic × manuscript type (thesis vs. article) (H2a-H2c), and by adding and testing the three interactions manuscript characteristic × actor (student vs. academic) (H3a-H3c) (see preregistration for the equations).” (p22, line 6)*

*The hypotheses corresponding to RQ 1-3 are explicated in the introduction, and from their formulation it is clear that they refer to main effects (R1) and interaction effects (R2-R3).*

Methods:
1. I found it confusing that PhD students seem to be considered as “researchers”, while the “student” category is reserve for bachelor and master students. I have no problem with this somewhat arbitrary category, but it should be made clear in the methods (until getting to Table 1, I was assuming that PhD candidates would be considered students).

*We addressed this by adding “PhD candidates were considered as academics rather than students” at the start of the methodology section.*

2. Sample size and statistical results can certainly interact in driving quality assessments (i.e. “could not explain the sex differences” in the vignette because of a p = 0.6 is a more appropriate conclusion when sample size/statistical power is high, but may be too strong a conclusion when it is low). Were interactions between the three manipulations tested in the first regression model? I assume they are present in the “full model”, but I could find no direct results concerning them, and I’d be curious about them.

*As we neither had hypotheses nor expectations on these interactions, and did not preregister these analyses, we did not test the four interaction effects of the three manuscript characteristics (i.e., S (sample size) × I (statistical significance), S × E (reporting error), I × E, S × I × E.*

*Generally, it is not recommended to test interactions, let alone higher-order interactions, when having no expectations on them or when the data do not suggest the presence of such interactions (see, e.g., the book of Keith (2006, p.137). ‘*Multiple regression and beyond’*). As main effects and first-order interactions provide no or at best weak evidence of any effect, we do not expect any higher-order interactions.*

3. Was the vignette presented before or after the survey? I got the impression that it came before, but given that one can influence the other it is important to make this clear.

*Yes, after, and we made this clear in the text. The abstract now reads “In the subsequent survey…” and in the design section “Participants of the vignette were subsequently asked in the survey to evaluate…”*

4. The “conduct” category in the survey is a bit of a hodgepodge. I don’t really see what “providing open data, materials, etc.” has to do with “achieving a high response rate” (which is independent of the researchers) or “achieving high statistical power” (which depends on items mentioned in the design section”). There is no way to go back on this now, but it may be worth mentioning in the limitations section.

*See also our response to comment 5 of R1. Note that the categories are always subjective to a certain extent, but that we also analyzed the manuscript characteristics individually, and not only at the domain or category level. We now state explicitly in the “Results” section:*

*“However, as characteristics within domains varied substantially, it is more meaningful to list those that were considered more important.” (p33, line 12)*

*Hence the domains or categories are now less important. Note that we already discuss the limitations of the manuscript characteristics in two paragraphs of the “discussion” section.*

Results:
1. For the vignette study, I don’t think it is very useful to look at the effect sizes for each factor in each group separately (Students, Academics/Thesis, Academics/Article), as is done in Table 4, without looking first at the main effect of these factors in the whole population (for which there is a much larger sample size and statistical power) – which only happens afterwards in the manuscript. Moreover, presenting these factors separately gives the impression of variation that may be just noise from these relatively small sample sizes (and nothing in the table allows one to evaluate this).
Please consider presenting the regression results first, and then presenting the effect sizes separately for each group (with p values for the interaction between manipulations and conditions, which currently appear in Table 6 but not in Table 4) to allow the reader to judge whether the variation is above what would be expected by chance.

*Table 4 presents the descriptive results corresponding to our hypotheses, with their effect sizes in Cohen’s d. By first presenting the descriptive statistics we follow the norm in several fields of psychology. Adding the rather well-known Cohen’s d effect size measures gives the reader a better idea of how large the effects are. You are right that the statistical power is increased by combined conditions but note that our hypotheses are formally tested by the regression analyses, of which the results are reported in Table 6. We can also remove Table 4 from the paper and only present Table 6. But then the reader would miss the descriptive statistics, and we believe this would be rather unfortunate.*

2. Please replace asterisks by exact p values whenever possible (e.g. Table 4).

*Done.*

3. Table 6 is quite hard to understand. I could eventually make out the meaning of each term, but terms such as “constant”, “sample”, “sig”, “error”,, “Sample*MT” etc. should be defined in the legend (or expressed in reader-friendly terms in the table itself).

*‘Constant’ has been replaced by intercept. ‘Sample’, ‘Sig’, and ‘Error’ and interactions with manuscript type (MT) and career phase (CP) are described in the note of the table. When used for the first time, the acronyms MT and CP are between brackets in the table itself (i.e., ‘Manuscript type (MT)’ and ‘Carreer phase (CP)’).*

4. Similarly, Table 7 is only comprehensible to the reader if he refers back to Table 3 (which specifies the meaning of T1, etc.). I think it would become much more reader friendly if the description of each item was included. If space is a problem, it’s probably worth including only the totals (as the differences between conditions are minor), leaving the six separate conditions only in Fig. 3, which provides a more intuitive way to look a the comparisons.

*Thank you, you are right. We added the labels of Table 3 to Table 7 as well and made the Table landscape.*

5. In Figure 3, the X axis should include the category to which the data refers to. Specially for the analysis and presentation category, it is quite annoying to have to count in order to discover which category each point refers to. Once more, brief descriptors would be more useful than T1, T2, etc., but space may be an issue in this case.

*Thank you, we agree. We added a short description on the x-axis.*

6. Table 8 could probably be omitted or moved to the supplementary material, as the close correlation across conditions can be visualized in Figure 3, and knowing the exact correlation value for each of them does not add much, and doesn’t really help to answer the research questions 6-13 (see major concern # 3)

*Here, we disagree. As we explained in our response to your ‘major concern 3’, we believe that the correlations provide an adequate and efficient way to demonstrate that the very same criteria are deemed most relevant for students and academics when assessing the quality of theses and articles.*

Discussion:
1. The last paragraph of the article is a confusing mix of different subjects and could be replaced by a more focused conclusions section.

*We agree. We deleted the first four sentences and the last, keeping the sentences on the importance of examining similar research questions in other fields and over time, also in the light of the increasing popularity of open science.*

Point issues:
1. Line 104 – there is a loose parenthesis.

*Thank you for spotting it, we removed it.*

2. Line 156 – “For example, the sample size of what students believe to be a large enough” – this sentence is odd (i.e. a large enough what?). Please revise.

*We changed the part of the sentence to “For example, what students believe may be a sufficient sample size (e.g., to have sufficient statistical power) might be influenced by sample sizes encountered in the literature studied in various courses.” (p8, line 12)*

3. Line 160 – When quoting numbers such as “over 90% of psychology researchers admitted having engaged in QRPs”, it’s worth stating more precisely what is considered under the “QRPs” umbrella.

*The list of QRPs is long, and as the paper is not focusing on QRPs we decided not to list the dozens of QRPs here. We provided four literature references instead.*

4. Line 175: “We did not only investigate the believes”. “Beliefs”, not believes.

*Thanks for spotting the error, we corrected it.*

5. Line 180: What the authors meant by “Statistical reporting error” was not clear to me at this point, perhaps it’s worth defining it better (e.g. “the presence of discrepancies in statistical values” or something like that).

*We added its definition at this point: “A statistical reporting error refers to an inconsistency between the reported test statistic, degrees of freedom, and p-value.” (p9, line 17)*

6. Line 206: “differs between students and academics”, not “from between”.

*Corrected, thanks!*

7. Box 1: The red color used to highlight the last manipulation makes it hard to read the text, consider changing to a lighter hue. Also, I’d argue that this is technically a figure and not a text box.

*Thanks. We changed the color to cyan. We see it as a text box, as we typed the text in a box within the manuscript.*

8. Line 458. “All 800 participants were asked to name three aspects they considered relevant for their evaluation of the manuscript quality.” – consider specifying “to the quality of the abstract presented in the vignette” to make it clear that the open responses concern the vignette and not manuscript quality in general.

*This is a misunderstanding. The survey concerns manuscripts in general, and not the abstract in the vignette. This is also not possible, as the abstract in the vignette only has about 200 words. Perhaps the misunderstanding is caused the word “the” preceding “manuscript quality” in the sentence you cite. We deleted this word “the” in that sentence.*

9. Discussion: “We expect reviewers consider sample size of higher importance than the participants in this study did, given that small sample size may be considered a red flag in quality assessment of an article, particularly in combination with a (barely) statistically significant outcome, which more likely represents a false positive.” I could not understand this statement. Do the expectations mentioned concern reviewers in general?

*In hindsight, we no longer strongly expect the statement to be true, and therefore deleted the sentence.*

10. Discussion: “In the current study, reviewers did not seem to be the main source of publication bias.” This is a weird statement: there is no publication decision being made un in the study, so it seems strange to talk about “publication bias”.

*We deleted this statement here, as well as in the abstract.*
