# Supplementary material for: The effect of manuscript characteristics on quality assessment of scientific manuscripts in psychology
Source: PLoS One. 2026 Sep 15;21(9):e0355938. doi: 10.1371/journal.pone.0355938 (PMC13577479; doi:10.1371/journal.pone.0355938)
Supplement: Supplementary file 5 [file pone.0355938.s003.docx]

**Response to comments on our revision of “Quality assessment of scientific manuscripts in peer review and education” (PONE-D-24-57377R1)**

*We thank the editor and the three reviewers for their comments. Below, we respond to each comment in italics, while using normal font for the comments of editor and reviewers.*

COMMENTS OF THE EDITOR

Dear Dr. van Assen,

Thank you for submitting your manuscript to PLOS ONE. After careful consideration, we feel that it has merit but does not fully meet PLOS ONE’s publication criteria as it currently stands. Therefore, we invite you to submit a revised version of the manuscript that addresses the points raised during the review process.

In your review, please provide thorough responses to all reviewers. Be sure to include additional details that address Reviewer 2's concerns about the correlations you describe. Ensure you comprehensively address every unresolved or remaining statistical issues they have identified.

*We addressed all issues below.*

Please submit your revised manuscript by Apr 21 2026 11:59PM. If you will need more time than this to complete your revisions, please reply to this message or contact the journal office at [plosone@plos.org](mailto:plosone@plos.org). When you're ready to submit your revision, log on to <https://www.editorialmanager.com/pone/> and select the 'Submissions Needing Revision' folder to locate your manuscript file.

Please include the following items when submitting your revised manuscript:

- A letter that responds to each point raised by the academic editor and reviewer(s). You should upload this letter as a separate file labeled 'Response to Reviewers'.
- A marked-up copy of your manuscript that highlights changes made to the original version. You should upload this as a separate file labeled 'Revised Manuscript with Track Changes'.
- An unmarked version of your revised paper without tracked changes. You should upload this as a separate file labeled 'Manuscript'.

If you would like to make changes to your financial disclosure, please include your updated statement in your cover letter. Guidelines for resubmitting your figure files are available below the reviewer comments at the end of this letter.

If applicable, we recommend that you deposit your laboratory protocols in protocols.io to enhance the reproducibility of your results. Protocols.io assigns your protocol its own identifier (DOI) so that it can be cited independently in the future. For instructions see: [https://journals.plos.org/plosone/s/submission-guidelines#loc-laboratory-protocols](https://track.editorialmanager.com/CL0/https:%2F%2Fjournals.plos.org%2Fplosone%2Fs%2Fsubmission-guidelines%23loc-laboratory-protocols/1/010f019cc6edcd31-dbfbcf72-d874-485e-80ac-8a6190c16386-000000/_XkU1zG3joSlHZb1VgF9lY03SW-V0cLvAeGVmvPXA5E=253). Additionally, PLOS ONE offers an option for publishing peer-reviewed Lab Protocol articles, which describe protocols hosted on protocols.io. Read more information on sharing protocols at [https://plos.org/protocols?utm_medium=editorial-email&utm_source=authorletters&utm_campaign=protocols](https://track.editorialmanager.com/CL0/https:%2F%2Fplos.org%2Fprotocols%3Futm_medium=editorial-email%26utm_source=authorletters%26utm_campaign=protocols/1/010f019cc6edcd31-dbfbcf72-d874-485e-80ac-8a6190c16386-000000/W4xlL5BpWJWedcexI1KTsB1u5E-r6DEcAu-J85-QSsc=253).

We look forward to receiving your revised manuscript.

Kind regards,

Sonia Vasconcelos, PhD
Academic Editor
PLOS One

Journal Requirements:

If the reviewer comments include a recommendation to cite specific previously published works, please review and evaluate these publications to determine whether they are relevant and should be cited. There is no requirement to cite these works unless the editor has indicated otherwise. 

Please review your reference list to ensure that it is complete and correct. If you have cited papers that have been retracted, please include the rationale for doing so in the manuscript text, or remove these references and replace them with relevant current references. Any changes to the reference list should be mentioned in the rebuttal letter that accompanies your revised manuscript. If you need to cite a retracted article, indicate the article’s retracted status in the References list and also include a citation and full reference for the retraction notice.

*Thank you. The reviewers did not ask us to add any references.*

OVERVIEW REVIEWERS’ COMMENTS

Reviewers' comments:

Reviewer's Responses to Questions

**Comments to the Author**
1. If the authors have adequately addressed your comments raised in a previous round of review and you feel that this manuscript is now acceptable for publication, you may indicate that here to bypass the “Comments to the Author” section, enter your conflict of interest statement in the “Confidential to Editor” section, and submit your "Accept" recommendation.

Reviewer #1: (No Response)

Reviewer #2: (No Response)

Reviewer #3: All comments have been addressed

*We like to note that the additional (statistical) reviewer, considers all comments of the two reviewers to be addressed in our revision.*

2. Is the manuscript technically sound, and do the data support the conclusions?

The manuscript must describe a technically sound piece of scientific research with data that supports the conclusions. Experiments must have been conducted rigorously, with appropriate controls, replication, and sample sizes. The conclusions must be drawn appropriately based on the data presented.

Reviewer #1: No

Reviewer #2: Partly

Reviewer #3: Yes

3. Has the statistical analysis been performed appropriately and rigorously?

Reviewer #1: No

Reviewer #2: Yes

Reviewer #3: Yes

*We are happy to read that the statistical Reviewer 3, who was asked for by Reviewer 1, considers our statistical analyses to have been performed appropriately and rigorously.*

4. Have the authors made all data underlying the findings in their manuscript fully available?

The [PLOS Data policy](http://track.editorialmanager.com/CL0/http:%2F%2Fwww.plosone.org%2Fstatic%2Fpolicies.action%23sharing/1/010f019cc6edcd31-dbfbcf72-d874-485e-80ac-8a6190c16386-000000/tto3GIbRp9towXWJlIv4Sqivow6p8w0yVHddXvSpQr0=253) requires authors to make all data underlying the findings described in their manuscript fully available without restriction, with rare exception (please refer to the Data Availability Statement in the manuscript PDF file). The data should be provided as part of the manuscript or its supporting information, or deposited to a public repository. For example, in addition to summary statistics, the data points behind means, medians and variance measures should be available. If there are restrictions on publicly sharing data—e.g. participant privacy or use of data from a third party—those must be specified.

Reviewer #1: Yes

Reviewer #2: Yes

Reviewer #3: Yes

5. Is the manuscript presented in an intelligible fashion and written in standard English?

PLOS ONE does not copyedit accepted manuscripts, so the language in submitted articles must be clear, correct, and unambiguous. Any typographical or grammatical errors should be corrected at revision, so please note any specific errors here.

Reviewer #1: No

Reviewer #2: Yes

Reviewer #3: Yes

6. Review Comments to the Author

Please use the space provided to explain your answers to the questions above. You may also include additional comments for the author, including concerns about dual publication, research ethics, or publication ethics. (Please upload your review as an attachment if it exceeds 20,000 characters)

REVIEWER 1

Dear authors,

thank you for the revision, but I find that you did not address my comments in the way I would want them, and so I advised the editor to get an extra statistical reviewer to weigh in on differences between my requests and your approaches.

*We again thank Reviewer 1 for their comments and their time to review our paper. Reviewer 3 considers our statistical analyses valid and your points addressed. We address Reviewer 3’s other points below.*

REVIEWER 2

The manuscript has addressed many of the concerns raised previously by both reviewers, especially concerning issues in the text and tables.

*Thank you. The revision took us considerable time, but because of it our paper also improved considerably, in our opinion.*

That said, I think some of the major concerns mentioned in the previous review remain unaddressed. I will focus this review on these main points.

1. I don’t think my main concern (major concern #1) has been addressed by the authors. They did include remarks that one should be careful of generalizing the conclusions based on evaluation of an abstract to evaluation of whole manuscripts or theses, which is appropriate. But they still fail to comment on the (to me, much more important) limitation that the conclusions relating to the vignette study are based on evaluation of a single, specific abstract that was presented to all participants, with a single, specific manipulation of each factor.
Thus, the analysis does not allow one to generalize the conclusions even to other abstracts or other manipulations. It may well be the case that, for different statistical errors, different p values, different results, or different abstract topics, the results found would be very different.

*We do not agree that ‘it may well be the case that’ rather slight changes in the manipulation may bring about very different results. In our experiences with replicating studies, slight changes in the manipulation cause at most slight (and sometimes to medium) changes in effect size.*

This is the point I was trying to get to in my previous review: I still believe it is a very important limitation, and it is still not mentioned anywhere in the discussion.

*Thank you for coming back to your major concern #1 again. We do actually agree with you that different manipulations may lead to different results. Hence, we changed the text in our manuscript in the following way, in the paragraph on the generalizability of our results (starting at the last sentence of p.38):*

*“And finally, at a more general level, other manipulations of statistical significance, sample size, and statistical reporting error may also lead to other results than we found in our vignette using an abstract of a scientific manuscript.”*

2. I still think that a correlation analysis between the responses of different groups of researchers (e.g. students vs. academics, those evaluating “theses” vs. those evaluating “articles” ) maps poorly to research questions 6 to 13, which purportedly aim to study differences between groups or conditions.
I am sensitive to the authors’ argument that studying the overall similarity of answers via correlations is more economical for presenting the results, as evaluating pairwise differences between answers for different topics would generate a lot of numbers to look at.

*We are glad to read that Reviewer 2 is sensitive to our argument that correlational analyses are more economical than pairwise differences. We also would like to stress again that*

*(i) the investigation of research questions 4 to 13 was exploratory as is also indicated in our preregistration, and*

*(ii) the strong correlations make clear that the order of responses is very similar across the categories in Figure 1 (i.e., thesis quality and thesis grading rated by both academics and students, and article quality, and article publishability rated by academics).*

But if the authors are only measuring correlations, they should be clear that the results mean that answers across groups and conditions are highly correlated, not that they are “the same”, as is used in the discussion (e.g. page 38, line 9, as well as multiple times in the responses to reviewers).

*Yes, good point. We adapted the text of our manuscript accordingly, replacing ‘the same’ with ‘similar’ (p.40, line 4) and ‘equal’ with ‘highly similar’ (p.40, line 10).*

There is still space for single items to vary considerably within a correlation of 0.9, and some differences in Table 7 appear to be quite unlikely to have arisen by chance given the sample size, even though no statistics are provided (e.g. preregistration has a quality rating of 4.89 for students vs. 3.70 for academics).

*Yes, these differences in scores are most apparent in Figure 3, as variation within each ‘clutter’.*

The authors may refrain from doing inferential statistics to evaluate these differences if they wish, but in this case they should (a) note that differences in individual items do occur between populations/conditions, perhaps citing the most obvious ones and (b) acknowledge in the discussion that the analysis is not really looking into differences between importance ratings as stated in the discussion, but rather assessing the overall similarity between the relative importance of different items. Alternatively, they may also actually evaluate differences in individual items using inferential statistics.

*Yes, we understand and agree with you.*

*The correlations assess the similarities in trends across questions (or ‘columns’ in Figure 3) for the six categories research-thesis-quality, research, research-thesis-grade, research-article-quality, research-article-publishability, student-thesis-quality, student-thesis-grade. But the correlations don’t say anything about differences in averages per question (i.e., within each ‘column’ in Figure 3). Although in this paper we were less interested in differences in average rated importance of manuscript characteristics, we now include in our main text some statements about these differences in averages (p.36, from line 17 onwards):*

*“Although correlations were very high, average importance ratings of manuscript characteristics sometimes differed across conditions, as can be seen in Figure 3 as well as Table 7. Focusing on differences of at least 0.5 standard deviation (((〖SD〗_1+〖SD〗_2)⁄2), using a large number of literature references (T4) was considered more important by students for the quality and grading of theses than by academics for the quality and publishability of articles. Interestingly, preregistration (D6) was rated by students as more important for the quality of a thesis than by academics for anything (all four conditions), and as more important by students for the grading of theses than by academics for the quality of an article.”*

3. I still think that the presentation of questions 4 to 13 in the introduction within a single text block is confusing. I’d strongly suggest that the authors list these individually, as they did for questions 1-3. Figure 1 is not immediately intuitive to the reader to fill that gap, and the brief legend does not help that much).

*As the investigation of research questions 4 to 13 was exploratory as is also indicated in our preregistration, and the explanation of the results of the statistical analyses to answer them was straightforward and short in the main text of our paper, we decided not to present and write them in detail in our introduction.*

*However, we agree that Figure 1 needs more explanation for all readers to understand. Hence, we expanded the legend in the following way (the first sentence is the same as in the original legend) (p.11, bottom):*

*“Figure 1*

*Overview of Explorative Research Questions of the Survey Study on the Evaluation of Scientific Manuscripts. Six different evaluations are made. Academics (top) evaluate both ‘quality’ and ‘grading’ of theses (left) and the ‘quality’ and ‘publishability’ of articles (right). Students (bottom) only evaluate the ‘quality’ and ‘grading’ of ’theses’ (left). Arrows 6-9 and 11-13 refer to correlations between two evaluations. For instance, and more specifically, arrow 9 corresponds to the correlation between the evaluated importance of 29 manuscript characteristics for ‘grading theses by academics’ and the evaluated importance of these same manuscript characteristics for the ‘publishability of articles by academics’. Arrow 10 refers to a difference between two correlations. The preregistration lists all research questions verbally.”*

*Note that, in the last sentence of the legend of Figure 1, we also refer to the preregistration that lists and describes each research question in words.*

4. If interactions between sample size and statistical significance in driving results were not tested (i.e. previous Methods point #2), this should be mentioned as a limitation in the discussion as well. I would certainly have an expectation that statistical significance may have different effects on quality judgments depending on sample size, as the appropriate interpretation for a given p value in terms of concluding about presence/absence of an effect should depend on the statistical power of the study.

*We estimated and tested the N×Significance interaction effect. The coefficient is not statistically significant: b = 0.17, t = 0.845, p = .40. We added these results to the results section (p.26, lines 16-17).*

*“Exploratively, we also tested the sample size × statistical significance interaction, but found no effect either (B = 0.17, t = 0.85, p = .398).”*

Minor points:
Table 6 – Career, not career

*Thanks for spotting that error, we corrected it.*

REVIEWER 3

Dear authors, I had some concerns. First, whether the study evaluated internal consistency using Cronbach's alpha or McDonald's omega . Did you create the instrument or, in this case, did you use it from a specific author and the information from the instrument? If the instrument was created by the authors, was there any type of validation by expert judgment, followed by a pilot study and, therefore, internal consistency and instrument validity using an AFC and an AFE?

*We did not use a scale for the first part, the vignette study. For the second part, the survey, as preregistered, we analyzed the data at the item-level for RQ4 and RQ5 (because KMO-indices were not sufficient). Also, for RQ6-RQ13 we correlated evaluated manuscript characteristics at the item-level. Hence, we did not use scales for answering our research questions and consequently did not compute Cronbach’s alpha (or McDonald’s omega).*

*We do compute averages, but mostly for comparison and illustration, but quickly continue with analyses at the item-level. More specifically, we wrote in our manuscript (p.32, from line 10 onwards):*

*“On average, theory ranked highest on importance, closely followed by analysis and presentation (0.2 SD lower), with even lower average scores for design (-0.5 SD) and conduct (-0.9 SD). However, as characteristics within domains varied substantially, it is more meaningful to list those that were considered more important.”*
