# Supplementary material for: The effect of manuscript characteristics on quality assessment of scientific manuscripts in psychology
Source: PLoS One. 2026 Sep 15;21(9):e0355938. doi: 10.1371/journal.pone.0355938 (PMC13577479; doi:10.1371/journal.pone.0355938)
Supplement: Peer review history

# Peer review: Decision Letter 0

**DOI:** 10.1371/journal.pone.0355938.r001

**Authors:** Sonia Vasconcelos

18 Mar 2025

Dear Dr. van Assen,

Thank you for submitting your manuscript to PLOS ONE. After careful consideration, we feel that it has merit but does not fully meet PLOS ONE’s publication criteria as it currently stands. Therefore, we invite you to submit a revised version of the manuscript that addresses the points raised during the review process.

**In your review, please provide detailed answers, particularly regarding the statistical analyses and the application of scale, domains, and scores, as discussed by both reviewers. Among Reviewer #2’s concerns is the use of frequentist analysis for most of the study without reasonable explanation. Please explain the reasoning behind choosing this statistical method for your hypotheses.**

**Both reviewers have also expressed skepticism about sample validation, recruitment, and data interpretation. Reviewer #1 is also concerned about how readers should interpret your scores and criteria to assess the quality of abstracts, manuscripts, and theses, as they do not necessarily reflect peer review practices.**

Please submit your revised manuscript by May 02 2025 11:59PM. If you will need more time than this to complete your revisions, please reply to this message or contact the journal office at plosone@plos.org. When you're ready to submit your revision, log on to https://www.editorialmanager.com/pone/ and select the 'Submissions Needing Revision' folder to locate your manuscript file.

- A rebuttal letter that responds to each point raised by the academic editor and reviewer(s). You should upload this letter as a separate file labeled 'Response to Reviewers'.
- A marked-up copy of your manuscript that highlights changes made to the original version. You should upload this as a separate file labeled 'Revised Manuscript with Track Changes'.
- An unmarked version of your revised paper without tracked changes. You should upload this as a separate file labeled 'Manuscript'.

If you would like to make changes to your financial disclosure, please include your updated statement in your cover letter. Guidelines for resubmitting your figure files are available below the reviewer comments at the end of this letter.

If applicable, we recommend that you deposit your laboratory protocols in protocols.io to enhance the reproducibility of your results. Protocols.io assigns your protocol its own identifier (DOI) so that it can be cited independently in the future. For instructions see: https://journals.plos.org/plosone/s/submission-guidelines#loc-laboratory-protocols. Additionally, PLOS ONE offers an option for publishing peer-reviewed Lab Protocol articles, which describe protocols hosted on protocols.io. Read more information on sharing protocols at https://plos.org/protocols?utm_medium=editorial-email&utm_source=authorletters&utm_campaign=protocols.

We look forward to receiving your revised manuscript.

Kind regards,

Sonia Vasconcelos, PhD

Academic Editor

PLOS ONE

**Journal Requirements:**

1. When submitting your revision, we need you to address these additional requirements.

Please ensure that your manuscript meets PLOS ONE's style requirements, including those for file naming. The PLOS ONE style templates can be found at

https://journals.plos.org/plosone/s/file?id=wjVg/PLOSOne_formatting_sample_main_body.pdf and

https://journals.plos.org/plosone/s/file?id=ba62/PLOSOne_formatting_sample_title_authors_affiliations.pdf

2. We note that the grant information you provided in the ‘Funding Information’ and ‘Financial Disclosure’ sections do not match.

When you resubmit, please ensure that you provide the correct grant numbers for the awards you received for your study in the ‘Funding Information’ section.

3. Thank you for stating the following financial disclosure:

Research Talent Grant of the Dutch Science Foundation (NWO).

Please state what role the funders took in the study.  If the funders had no role, please state: "The funders had no role in study design, data collection and analysis, decision to publish, or preparation of the manuscript."

If this statement is not correct you must amend it as needed.

Please include this amended Role of Funder statement in your cover letter; we will change the online submission form on your behalf.

4. Please note that your Data Availability Statement is currently missing the repository name and/or the DOI/accession number of each dataset. If your manuscript is accepted for publication, you will be asked to provide these details on a very short timeline. We therefore suggest that you provide this information now, though we will not hold up the peer review process if you are unable.

5. Your ethics statement should only appear in the Methods section of your manuscript. If your ethics statement is written in any section besides the Methods, please move it to the Methods section and delete it from any other section. Please ensure that your ethics statement is included in your manuscript, as the ethics statement entered into the online submission form will not be published alongside your manuscript.

6. We note that you have referenced “Cooper H, DeNeve K, Charlton K.”  which has currently not yet been accepted for publication. Please remove this from your References and amend this to state in the body of your manuscript: (ie “Cooper H, DeNeve K, Charlton K.” [Submitted]”) as detailed online in our guide for authors

http://journals.plos.org/plosone/s/submission-guidelines#loc-reference-style

7. Please include captions for your Supporting Information files at the end of your manuscript, and update any in-text citations to match accordingly. Please see our Supporting Information guidelines for more information: http://journals.plos.org/plosone/s/supporting-information.

[Note: HTML markup is below. Please do not edit.]

Reviewers' comments:

Reviewer's Responses to Questions

**Comments to the Author**

1. Is the manuscript technically sound, and do the data support the conclusions?

Reviewer #1: No

Reviewer #2: Partly

**********

2. Has the statistical analysis been performed appropriately and rigorously?

Reviewer #1: No

Reviewer #2: No

**********

3. Have the authors made all data underlying the findings in their manuscript fully available?

The PLOS Data policy

Reviewer #1: Yes

Reviewer #2: Yes

**********

4. Is the manuscript presented in an intelligible fashion and written in standard English?

Reviewer #1: Yes

Reviewer #2: Yes

**********

**Reviewer #1:**  Thank you for the opportunity to review this manuscript. The following are my recommendations for its improvement:

Title

1. As this is not about evaluation of actual manuscripts, I would suggest changing the title to: Exploring students and researchers rating of the quality of manuscripts

Abstract

1. Please include information on the quality scores given to vignettes, and quantify the influence of the sample size you mention. Additionally, specify if these were actual existing abstracts/manuscripts, or created for this experiment

2. These results suggest that researchers’ assessments of manuscript quality are not 24 responsible for publication bias. – I disagree this study was not designed to assess publication bias.

3. “are already adopted” – I find this conclusion wrong, this might indicate that science educations implies certain standardized quality considerations. I do not find adopted as a proper word in this context. We know from rant review, that when we specify domains and provide concrete ratings, researcher can apply those, same as teachers in grading students or their coursework. Why would researchers need to adopt anything, when they are not ask to rate manuscripts in this way in everyday research publishing or review.

Introduction

1. I believe authors have to discuss the influence of registered reports on the reporting of non-significant results, and current data on this, similarly they can comment on the results of clinical trials that have to be registered by law – i.e. researchers may chose to pursue studies for which their pilot analysis already indicate potential positive effect.

Peer Review

1. There are approximately 33,100 peer-reviewed – Please provide citation for this, current estimates are around 70,000 using Open Alex data. Similarly we have more than 6 million articles published per year, using OpenAlex or Dimensions

2. As the authors stated “we focus on the question which manuscript characteristics are most important for manuscript quality rating that might differ between reviewers and even between manuscripts.” – In regards to this I would greatly revise the current introduction and peer review section, and provide literature review on published papers that aimed to assess manuscript or peer review quality, and report on methods used to do so, and domains assessed in previous research.

3. “also tested what influenced their quality assessment” – was this testing part of the original study when the original question was asked? If not, this should be clearly stated.

Participants

1. Please report what was the original plan for not meeting the sample size. You stated you needed at least 787 participants, and you obtained 687 – why did you not conduct another or several more rounds of invites?

2. “ even when their main field of research was not within a research field in psychology” – Please spicy if this was originally planed like this, or were you aiming to collect responses only from individuals with primary field of psychology.

3. Due to later statement “in which psychology research field they primarily worked”, I would appreciate a sub-analysis of results, with those not primarily working in psychology being excluded, and compared to results of those that do.

4. Table 1, I would suggest that the reporting is changesdto N (%) with 0 decimal points, or on the reverse order, the use of n=, seems unnecessary.

5. Table 3 – I dislike the phrasing - Using a large number of literature references., I would find this to have a QRP associated with it, Using relevant lit. references seems more important than the number. Similarly phrasing of using complex design and using complex analyses, seem out of place in the list. The researchers should be aware of the pyramid of evidence, which of those do the drafters consider to the complex? Why Is using complex analysis in C, when using appropriate analysis is in A – this seems to be bad design of the scale and domains. What reliability testing has been done to confirm that these items belong under the same domains? It seems evident from the way respondents answered the question on what influences the ratings, that they might not adhere to the domains your teams have devised. In a registered report or protocol with statistical analysis plan predefined, is using the appropriate statistics part of design or only of analysis? Why is providing code or data part of conduct and not of presentation? Why is describing the sample part of design and not presentation? You had a planned sample, and you did not reach it. The sentence “Clearly describing the sample population” – can only occur after analysis of the results, so it can not be part of design as Table 3 would imply. In short, please indicate in the survey itself, were respondents presented the sentences with the domains or only sentences? I was not surprised to read later that the KMO scores were low, as these domains to me do not meet face validity.

6. Since hypotheses 1c, 2c and 3c indicated no effect, we evaluated these hypotheses using Bayesian statistics. – Please indicate if this analysis was preregistered? If not, this should be moved to appendix.

7. I would suggest putting content coding as a subtitle in the analysis section

Results

1. Please indicate in the stat. section if the quality rating data was normally distributed to report M and SD, instead of Md and IQR?

2. Reporting of aspects – would it be possible for you to showcase the first choice of the most important aspect of participants, then the second, then the third? I am not sure that the coding you applied matches the domain coding of researchers themselves. You separated Reporting of statistical results, statistical significance, and statistical reporting of error, from results reporting. And yet in Table 3, these items fall under domains of conduct or analysis and presentation. I would have expected your own top domains to be identical. You state “other (e.g. preregistration, open data)”, but in Table 3 this is part of conduct, which I disagree with conceptually, to me this is part of presentation, but at least you should apply your own consistent coding and justification behind it.

3. Results style – As you had 13 research questions, please rewrite the results in the order of those questions, with appropriate subtitles and report them in that order. I would highly recommend, that you start with, results for our 13 predefined research questions are provided in Table X or Box X, which would list each question, the answer to it, as well as strength of evidence rating for it, with indication or planned vs additional analysis to explore/confirm the RQ.

4. “As the items in the survey did not correlate sufficiently” – Please start this section first with a little explanation. To test X, we conducted KMO analysis which indicated that the prespecified domains were no…. As preplaned, we then analysed data at the item level. “the average rating of each category “ – why is this shown, when you have evidence that they do not belong to the same category?

5. “Theory was rated to be the most important” – I am not sure that this is the proper way to interpret these results. As you decided on item level presentation, list the items with most. One of the design items had a very high score too, and readers should know which of the individual items statistically differ in scores from which items? In regards to the vignette, where answers indicated that design is by far the most important aspect (when asked to list 3 aspects), we as readers should know if this second type of rating had stat. sign difference between which individual level items.

6. Table 7 – average domain level scores should be deleted, due to KMO scoring indicating these are not the same domain. Those that had appropriate scores, can be considered for average score, if you feel that appropriate.

Discussion

1. I will refrain from detailed comments on discussion until the above are clarified. I would however advise authors to make it very clear that researchers are not normally asked to rate the quality of abstract/manuscripts/thesis using the scale presented in this domain. And the peer review of the manuscripts does not work in regards of improving the initial given score, to then a higher score, as many aspects of the design can rarely be changed. So I would suggest emphasizing in the introduction and discussion, that in this exercise of trying to score manuscripts, what was found. Also, we have no test-rest scores for this, and we are not sure, that the same researchers would give the same score or not change their criteria when asked to do these kind of tasks regularly.

In hopes the above can help improve the readability and presentation of the results.

**Reviewer #2:**  The manuscript describes an interesting empirical attempt at studying some aspects of how reviewers evaluate articles or theses. Unfortunately, there are some important limitations that are not adequately addressed by the authors. I also have some issues concerning the statistical analyses for some of the hypotheses under study.

Major concerns:

1. As far as I could understand, the entire vignette study was based on a single abstract, with three elements (sample size, statistical result and reporting error) that varied between two different categories.

This poses an important restriction on any generalizations that can be drawn from the study, as one cannot generalize from this specific case how much judgments of quality by researchers or students are affected by these three factors in general. For instance, this is only one example of a reporting error (which may not be particular evident for most people): other reporting errors may be more easily noticed and have a much larger impact on quality judgments. Similarly, studies with different designs or topics may have their quality assessments more or less affected by sample size or statistical results.

Generalizing from the experimental study to general statements concerning what researchers take into account when evaluating manuscript would likely require multiple examples, which would ideally be entered into a statistical model as random variables (for more on this issue, see Yarkoni, 2020 - https://doi.org/10.1017/s0140525x20001685).

This seems to me the most important limitation on what can be concluded from the study – however, it does not seem to be mentioned at all in the limitations section. The impossibility of generalizing from this single abstract to manuscripts/theses in general should be clearly stated both in the introduction and discussion, and taken into account when presenting the results.

This is particularly important for the statistical reporting error variable, the detection of which seems to require that participants would be able to spontaneously notice that a p value does not correspond to a given t value (a correspondence which most researchers are not used to doing without the use of software or tables). I would thus strongly avoid making any kind of generalization about how much researchers perceive or value reporting errors based on this example alone.

2. The recruitment of researchers and students is done through very different methods: thus, any statistical comparisons may be reflecting differences in the populations induced by the recruitment methods, rather than the different career stages. Particularly, the fact that students were recruited through a method that was much more dependent on the personal contacts of the researchers raises concern that this may have selected a population that was more familiar with discussions on open science/reproducibility than the general population of researchers recruited through the Web of Science.

This should also be explicitly mentioned in the discussion section and in any comparisons drawn between researchers and students throughout the manuscript.

3. Research questions 6 to 13 seem not to have been addressed by the statistical analysis at all. Although the formulation of these questions is rather haphazard in the manuscript (which makes it difficult to understand some of them), the authors state that they will “study differences in the relevance of the different characteristics” when assessing quality vs. publishability, theses vs. articles, etc.

However, the only statistical analyses presented for these findings are correlations between the answers in the different conditions (academics vs. students, articles vs. theses, quality vs. grading/publishability). These do not test for the presence of differences between these conditions at all, as the presented p values for correlations test the (highly unlikely) null hypothesis that there would be no correlation at all between answers in both conditions, and don’t answer the intended question of whether there are differences in the assessment of different features between the conditions at the population level.

To answer these questions, a direct statistical comparison between the rating of different items in different conditions would be needed, in order to test the null hypothesis that there is no association between conditions and survey answers. If the authors feel that the exploratory nature of the questions and the large number of items/conditions do not warrant formal significance testing, this is fine. However, it would still be useful to present p values or confidence intervals for differences. Moreover (and perhaps more importantly), statements such as “This suggests that academics uphold the same quality criteria for master’s theses as for manuscripts submitted for publication” are clearly not warranted based on a correlation analysis alone.

4. I find it quite strange to use traditional frequentist analyses for most of the article, but a Bayesian analysis for hypothesis 1c/2c/3c. If I understood correctly, this was done because the authors’ hypothesis was that there would be no effect, and thus they wanted to provide evidence in favor of the null hypothesis.

Doing this through Bayesian analyses, however, eliminates the commensurability between the analyses used for different hypotheses, as the conclusions that can be drawn from Bayesian vs. frequentist approaches are fundamentally different. The paper would thus be more coherent if either Bayesian or frequentist analyses (or both) were used for all hypotheses.

Arguing in favor of the lack of a large effect shouldn’t be an obstacle to use frequentist analysis: if the authors want to evaluate evidence against an effect, they can use traditional equivalence testing for this to remain within the framework used in the rest of the article. Conversely, there is no problem in using Bayesian analysis to test directional hypotheses such as 1a/b, 2a/b and 3a/b.

Minor concerns:

General:

1. Figure legends seem to be missing from the manuscript file altogether (or at least I could not find them).

2. For some reason, line numbers disappear after page 29.

Introduction:

1. The introduction is very long, with a lot of general sections about peer review that frequently touches on issues that are general knowledge for most scientists and are sometimes not directly related to the research questions.

I personally found this literature review quite interesting and thorough, but also somewhat superfluous. This is a matter of style and the authors do not need to change it, but I would consider shortening the introduction to make the paper more readable.

Study hypotheses:

1. The exploratory research questions (RQ4-RQ13) are formulated haphazardly and are hard to understand clearly either from the text or from Figure 1, which is hardly self-explanatory. Please consider formulating them one by one, as was done for the first research questions. Also note that RQ4 and 5 are not mentioned in the figure.

2. The correspondence between statistical analyses and hypotheses is also not clear. It seems to me that RQ2 and RQ3 are being tested through the interaction terms in the regression models, for example but it would be useful to state this clearly in the hypothesis or methods section.

Methods:

1. I found it confusing that PhD students seem to be considered as “researchers”, while the “student” category is reserve for bachelor and master students. I have no problem with this somewhat arbitrary category, but it should be made clear in the methods (until getting to Table 1, I was assuming that PhD candidates would be considered students).

2. Sample size and statistical results can certainly interact in driving quality assessments (i.e. “could not explain the sex differences” in the vignette because of a p = 0.6 is a more appropriate conclusion when sample size/statistical power is high, but may be too strong a conclusion when it is low). Were interactions between the three manipulations tested in the first regression model? I assume they are present in the “full model”, but I could find no direct results concerning them, and I’d be curious about them.

3. Was the vignette presented before or after the survey? I got the impression that it came before, but given that one can influence the other it is important to make this clear.

4. The “conduct” category in the survey is a bit of a hodgepodge. I don’t really see what “providing open data, materials, etc.” has to do with “achieving a high response rate” (which is independent of the researchers) or “achieving high statistical power” (which depends on items mentioned in the design section”). There is no way to go back on this now, but it may be worth mentioning in the limitations section.

Results:

1. For the vignette study, I don’t think it is very useful to look at the effect sizes for each factor in each group separately (Students, Academics/Thesis, Academics/Article), as is done in Table 4, without looking first at the main effect of these factors in the whole population (for which there is a much larger sample size and statistical power) – which only happens afterwards in the manuscript. Moreover, presenting these factors separately gives the impression of variation that may be just noise from these relatively small sample sizes (and nothing in the table allows one to evaluate this).

Please consider presenting the regression results first, and then presenting the effect sizes separately for each group (with p values for the interaction between manipulations and conditions, which currently appear in Table 6 but not in Table 4) to allow the reader to judge whether the variation is above what would be expected by chance.

2. Please replace asterisks by exact p values whenever possible (e.g. Table 4).

3. Table 6 is quite hard to understand. I could eventually make out the meaning of each term, but terms such as “constant”, “sample”, “sig”, “error”,, “Sample*MT” etc. should be defined in the legend (or expressed in reader-friendly terms in the table itself).

4. Similarly, Table 7 is only comprehensible to the reader if he refers back to Table 3 (which specifies the meaning of T1, etc.). I think it would become much more reader friendly if the description of each item was included. If space is a problem, it’s probably worth including only the totals (as the differences between conditions are minor), leaving the six separate conditions only in Fig. 3, which provides a more intuitive way to look a the comparisons.

5. In Figure 3, the X axis should include the category to which the data refers to. Specially for the analysis and presentation category, it is quite annoying to have to count in order to discover which category each point refers to. Once more, brief descriptors would be more useful than T1, T2, etc., but space may be an issue in this case.

6. Table 8 could probably be omitted or moved to the supplementary material, as the close correlation across conditions can be visualized in Figure 3, and knowing the exact correlation value for each of them does not add much, and doesn’t really help to answer the research questions 6-13 (see major concern # 3)

Discussion:

1. The last paragraph of the article is a confusing mix of different subjects and could be replaced by a more focused conclusions section.

Point issues:

1. Line 104 – there is a loose parenthesis.

2. Line 156 – “For example, the sample size of what students believe to be a large enough” – this sentence is odd (i.e. a large enough what?). Please revise.

3. Line 160 – When quoting numbers such as “over 90% of psychology researchers admitted having engaged in QRPs”, it’s worth stating more precisely what is considered under the “QRPs” umbrella.

4. Line 175: “We did not only investigate the believes”. “Beliefs”, not believes.

5. Line 180: What the authors meant by “Statistical reporting error” was not clear to me at this point, perhaps it’s worth defining it better (e.g. “the presence of discrepancies in statistical values” or something like that).

6. Line 206: “differs between students and academics”, not “from between”.

7. Box 1: The red color used to highlight the last manipulation makes it hard to read the text, consider changing to a lighter hue. Also, I’d argue that this is technically a figure and not a text box.

8. Line 458. “All 800 participants were asked to name three aspects they considered relevant for their evaluation of the manuscript quality.” – consider specifying “to the quality of the abstract presented in the vignette” to make it clear that the open responses concern the vignette and not manuscript quality in general.

9. Discussion: “We expect reviewers consider sample size of higher importance than the participants in this study did, given that small sample size may be considered a red flag in quality assessment of an article, particularly in combination with a (barely) statistically significant outcome, which more likely represents a false positive.” I could not understand this statement. Do the expectations mentioned concern reviewers in general?

10. Discussion: “In the current study, reviewers did not seem to be the main source of publication bias.” This is a weird statement: there is no publication decision being made un in the study, so it seems strange to talk about “publication bias”.

**********

what does this mean?). If published, this will include your full peer review and any attached files.

If you choose “no”, your identity will remain anonymous but your review may still be made public.

**Do you want your identity to be public for this peer review?**  For information about this choice, including consent withdrawal, please see our Privacy Policy

Reviewer #1: **Yes:**  Mario Malicki

Reviewer #2: **Yes:**  Olavo B. Amaral

**********

[NOTE: If reviewer comments were submitted as an attachment file, they will be attached to this email and accessible via the submission site. Please log into your account, locate the manuscript record, and check for the action link "View Attachments". If this link does not appear, there are no attachment files.]

While revising your submission, please upload your figure files to the Preflight Analysis and Conversion Engine (PACE) digital diagnostic tool, https://pacev2.apexcovantage.com/. PACE helps ensure that figures meet PLOS requirements. To use PACE, you must first register as a user. Registration is free. Then, login and navigate to the UPLOAD tab, where you will find detailed instructions on how to use the tool. If you encounter any issues or have any questions when using PACE, please email PLOS at figures@plos.org. Please note that Supporting Information files do not need this step.

# Author comment: Author response to Decision Letter 1

**DOI:** 10.1371/journal.pone.0355938.r002

11 Sep 2025

See our response letter.

Attachment Submitted filename: response letter PLOS ONE.docx

# Peer review: Decision Letter 1

**DOI:** 10.1371/journal.pone.0355938.r003

**Authors:** Sonia Vasconcelos

7 Mar 2026

Dear Dr. van Assen,

Thank you for submitting your manuscript to PLOS ONE. After careful consideration, we feel that it has merit but does not fully meet PLOS ONE’s publication criteria as it currently stands. Therefore, we invite you to submit a revised version of the manuscript that addresses the points raised during the review process.

Please submit your revised manuscript by Apr 21 2026 11:59PM. If you will need more time than this to complete your revisions, please reply to this message or contact the journal office at plosone@plos.org. When you're ready to submit your revision, log on to https://www.editorialmanager.com/pone/ and select the 'Submissions Needing Revision' folder to locate your manuscript file.

- A letter that responds to each point raised by the academic editor and reviewer(s). You should upload this letter as a separate file labeled 'Response to Reviewers'.
- A marked-up copy of your manuscript that highlights changes made to the original version. You should upload this as a separate file labeled 'Revised Manuscript with Track Changes'.
- An unmarked version of your revised paper without tracked changes. You should upload this as a separate file labeled 'Manuscript'.

If applicable, we recommend that you deposit your laboratory protocols in protocols.io to enhance the reproducibility of your results. Protocols.io assigns your protocol its own identifier (DOI) so that it can be cited independently in the future. For instructions see: https://journals.plos.org/plosone/s/submission-guidelines#loc-laboratory-protocols. Additionally, PLOS ONE offers an option for publishing peer-reviewed Lab Protocol articles, which describe protocols hosted on protocols.io. Read more information on sharing protocols at https://plos.org/protocols?utm_medium=editorial-email&utm_source=authorletters&utm_campaign=protocols.

We look forward to receiving your revised manuscript.

Kind regards,

Sonia Vasconcelos, PhD

Academic Editor

PLOS One

Journal Requirements:

If the reviewer comments include a recommendation to cite specific previously published works, please review and evaluate these publications to determine whether they are relevant and should be cited. There is no requirement to cite these works unless the editor has indicated otherwise.

Please review your reference list to ensure that it is complete and correct. If you have cited papers that have been retracted, please include the rationale for doing so in the manuscript text, or remove these references and replace them with relevant current references. Any changes to the reference list should be mentioned in the rebuttal letter that accompanies your revised manuscript. If you need to cite a retracted article, indicate the article’s retracted status in the References list and also include a citation and full reference for the retraction notice.

[Note: HTML markup is below. Please do not edit.]

Reviewers' comments:

Reviewer's Responses to Questions

**Comments to the Author**

Reviewer #1: (No Response)

Reviewer #2: (No Response)

Reviewer #3: All comments have been addressed

**********

2. Is the manuscript technically sound, and do the data support the conclusions?

Reviewer #1: No

Reviewer #2: Partly

Reviewer #3: Yes

**********

3. Has the statistical analysis been performed appropriately and rigorously?

Reviewer #1: No

Reviewer #2: Yes

Reviewer #3: Yes

**********

4. Have the authors made all data underlying the findings in their manuscript fully available?

The PLOS Data policy

Reviewer #1: Yes

Reviewer #2: Yes

Reviewer #3: Yes

**********

5. Is the manuscript presented in an intelligible fashion and written in standard English?

Reviewer #1: No

Reviewer #2: Yes

Reviewer #3: Yes

**********

Reviewer #1: Dear authors,

thank you for the revision, but I find that you did not address my comments in the way I would want them, and so I advised the editor to get an extra statistical reviewer to weigh in on differences between my requests and your approaches.

Reviewer #2: The manuscript has addressed many of the concerns raised previously by both reviewers, especially concerning issues in the text and tables. That said, I think some of the major concerns mentioned in the previous review remain unaddressed. I will focus this review on these main points.

1. I don’t think my main concern (major concern #1) has been addressed by the authors. They did include remarks that one should be careful of generalizing the conclusions based on evaluation of an abstract to evaluation of whole manuscripts or theses, which is appropriate. But they still fail to comment on the (to me, much more important) limitation that the conclusions relating to the vignette study are based on evaluation of a single, specific abstract that was presented to all participants, with a single, specific manipulation of each factor.

Thus, the analysis does not allow one to generalize the conclusions even to other abstracts or other manipulations. It may well be the case that, for different statistical errors, different p values, different results, or different abstract topics, the results found would be very different. This is the point I was trying to get to in my previous review: I still believe it is a very important limitation, and it is still not mentioned anywhere in the discussion.

2. I still think that a correlation analysis between the responses of different groups of researchers (e.g. students vs. academics, those evaluating “theses” vs. those evaluating “articles” ) maps poorly to research questions 6 to 13, which purportedly aim to study differences between groups or conditions.

I am sensitive to the authors’ argument that studying the overall similarity of answers via correlations is more economical for presenting the results, as evaluating pairwise differences between answers for different topics would generate a lot of numbers to look at. But if the authors are only measuring correlations, they should be clear that the results mean that answers across groups and conditions are highly correlated, not that they are “the same”, as is used in the discussion (e.g. page 38, line 9, as well as multiple times in the responses to reviewers). There is still space for single items to vary considerably within a correlation of 0.9, and some differences in Table 7 appear to be quite unlikely to have arisen by chance given the sample size, even though no statistics are provided (e.g. preregistration has a quality rating of 4.89 for students vs. 3.70 for academics).

The authors may refrain from doing inferential statistics to evaluate these differences if they wish, but in this case they should (a) note that differences in individual items do occur between populations/conditions, perhaps citing the most obvious ones and (b) acknowledge in the discussion that the analysis is not really looking into differences between importance ratings as stated in the discussion, but rather assessing the overall similarity between the relative importance of different items. Alternatively, they may also actually evaluate differences in individual items using inferential statistics.

3. I still think that the presentation of questions 4 to 13 in the introduction within a single text block is confusing. I’d strongly suggest that the authors list these individually, as they did for questions 1-3. Figure 1 is not immediately intuitive to the reader to fill that gap, and the brief legend does not help that much).

4. If interactions between sample size and statistical significance in driving results were not tested (i.e. previous Methods point #2), this should be mentioned as a limitation in the discussion as well. I would certainly have an expectation that statistical significance may have different effects on quality judgments depending on sample size, as the appropriate interpretation for a given p value in terms of concluding about presence/absence of an effect should depend on the statistical power of the study.

Minor points:

Table 6 – Career, not carreer

Reviewer #3: Dear authors, I had some concerns. First, whether the study evaluated internal consistency using Cronbach's alpha or McDonald's omega . Did you create the instrument or, in this case, did you use it from a specific author and the information from the instrument? If the instrument was created by the authors, was there any type of validation by expert judgment, followed by a pilot study and, therefore, internal consistency and instrument validity using an AFC and an AFE?

**********

what does this mean?). If published, this will include your full peer review and any attached files.

If you choose “no”, your identity will remain anonymous but your review may still be made public.

**Do you want your identity to be public for this peer review?**  For information about this choice, including consent withdrawal, please see our Privacy Policy

Reviewer #1: **Yes:**  Mario Malicki

Reviewer #2: **Yes:**  Olavo B. Amaral

Reviewer #3: No

**********

[NOTE: If reviewer comments were submitted as an attachment file, they will be attached to this email and accessible via the submission site. Please log into your account, locate the manuscript record, and check for the action link "View Attachments". If this link does not appear, there are no attachment files.]

To ensure your figures meet our technical requirements, please review our figure guidelines: https://journals.plos.org/plosone/s/figures

You may also use PLOS’s free figure tool, NAAS, to help you prepare publication quality figures: https://journals.plos.org/plosone/s/figures#loc-tools-for-figure-preparation.

NAAS will assess whether your figures meet our technical requirements by comparing each figure against our figure specifications.

# Author comment: Author response to Decision Letter 2

**DOI:** 10.1371/journal.pone.0355938.r004

22 Apr 2026

See the included document

Attachment Submitted filename: response letter PLOS ONE revision_HA.docx

# Peer review: Decision Letter 2

**DOI:** 10.1371/journal.pone.0355938.r005

**Authors:** Sonia Vasconcelos

10 Jul 2026

Dear Dr. van Assen,

Please submit your revised manuscript by Aug 24 2026 11:59PM. If you will need more time than this to complete your revisions, please reply to this message or contact the journal office at plosone@plos.org. When you're ready to submit your revision, log on to https://www.editorialmanager.com/pone/ and select the 'Submissions Needing Revision' folder to locate your manuscript file.

- A letter that responds to each point raised by the academic editor and reviewer(s). You should upload this letter as a separate file labeled 'Response to Reviewers'.
- A marked-up copy of your manuscript that highlights changes made to the original version. You should upload this as a separate file labeled 'Revised Manuscript with Track Changes'.
- An unmarked version of your revised paper without tracked changes. You should upload this as a separate file labeled 'Manuscript'.

If you would like to make changes to your financial disclosure, please include your updated statement in your cover letter. Guidelines for resubmitting your figure files are available below the reviewer comments at the end of this letter.

If applicable, we recommend that you deposit your laboratory protocols in protocols.io to enhance the reproducibility of your results. Protocols.io assigns your protocol its own identifier (DOI) so that it can be cited independently in the future. For instructions see: https://journals.plos.org/plosone/s/submission-guidelines#loc-laboratory-protocols. Additionally, PLOS ONE offers an option for publishing peer-reviewed Lab Protocol articles, which describe protocols hosted on protocols.io. Read more information on sharing protocols at https://plos.org/protocols?utm_medium=editorial-email&utm_source=authorletters&utm_campaign=protocols.

As the corresponding author, your ORCID iD is verified in the submission system and will appear in the published article. PLOS supports the use of ORCID, and we encourage all coauthors to register for an ORCID iD and use it as well. Please encourage your coauthors to verify their ORCID iD within the submission system before final acceptance, as unverified ORCID iDs will not appear in the published article. *Only*  the individual author can complete the verification step; PLOS staff *cannot*  verify ORCID iDs on behalf of authors.

We look forward to receiving your revised manuscript.

Kind regards,

Sonia Vasconcelos, PhD

Academic Editor

PLOS One

**Journal Requirements:**

1. If the reviewer comments include a recommendation to cite specific previously published works, please review and evaluate these publications to determine whether they are relevant and should be cited. There is no requirement to cite these works unless the editor has indicated otherwise.

2. Please review your reference list to ensure that it is complete and correct. If you have cited papers that have been retracted, please include the rationale for doing so in the manuscript text, or remove these references and replace them with relevant current references. Any changes to the reference list should be mentioned in the rebuttal letter that accompanies your revised manuscript. If you need to cite a retracted article, indicate the article’s retracted status in the References list and also include a citation and full reference for the retraction notice.

[Note: HTML markup is below. Please do not edit.]

Reviewers' comments:

Reviewer's Responses to Questions

**Comments to the Author**

Reviewer #2: (No Response)

Reviewer #3: All comments have been addressed

**********

2. Is the manuscript technically sound, and do the data support the conclusions?

Reviewer #2: Partly

Reviewer #3: Yes

**********

3. Has the statistical analysis been performed appropriately and rigorously?

Reviewer #2: Yes

Reviewer #3: Yes

**********

4. Have the authors made all data underlying the findings in their manuscript fully available?

The PLOS Data policy

Reviewer #2: Yes

Reviewer #3: Yes

**********

5. Is the manuscript presented in an intelligible fashion and written in standard English?

Reviewer #2: Yes

Reviewer #3: Yes

**********

Reviewer #2: I found the comments from my last review to have been at least partially addressed.

I do note that I strongly disagree with the authors’ statement in the response to reviewers that different statistical errors, topics or results would be “slight changes in the manipulation” and thus unlikely to change the observed effect. Statistical errors range from subtle to obvious, and their detectability (which is a prerequisite for taking them into account in the evaluation of an abstract) should be expected to vary accordingly. I do think that the one used in the vignette is on the subtle side, and I’d thus put much more emphasis on this limitation when discussing the results. But I think that this will be clear to the reader from the description of methods and results, so I won’t be fighting the authors further on this issue.

That said, I have found a problem which I had overlooked in previous reviews, which I think is quite important and does have to be fixed before publication. Although the study was entirely conducted with psychology students and researchers, the fact that they come from this research field is nowhere to be found either in the title or abstract, which only mention “students” and “academics” with no information on their background. I’d thus strongly advise the editor and authors to mention this both in the title (e.g. “The effect of manuscript characteristics on quality assessment of scientific manuscripts in psychology”) and abstract (e.g. “…psychology academics give of articles submitted for publication, and psychology academics and students…”).

Reviewer #3: Dear reviewers, based on the respective reviews that have been conducted.

Please review the document carefully to ensure there are no duplicated points at the end of paragraphs, especially in the abstract.

Please review the document again to verify that all the values mentioned are correctly described in the abstract.

Please verify that the formatting is correct (spacing, punctuation, and consistency).

We wish you the best of luck in advance for your contribution to the scientific community.

**********

what does this mean?). If published, this will include your full peer review and any attached files.

If you choose “no”, your identity will remain anonymous but your review may still be made public.

**Do you want your identity to be public for this peer review?**  For information about this choice, including consent withdrawal, please see our Privacy Policy

Reviewer #2: **Yes:**  Olavo B. Amaral

Reviewer #3: No

**********

[NOTE: If reviewer comments were submitted as an attachment file, they will be attached to this email and accessible via the submission site. Please log into your account, locate the manuscript record, and check for the action link "View Attachments". If this link does not appear, there are no attachment files.]

To ensure your figures meet our technical requirements, please review our figure guidelines: https://journals.plos.org/plosone/s/figures

You may also use PLOS’s free figure tool, NAAS, to help you prepare publication quality figures: https://journals.plos.org/plosone/s/figures#loc-tools-for-figure-preparation.

NAAS will assess whether your figures meet our technical requirements by comparing each figure against our figure specifications.

# Author comment: Author response to Decision Letter 3

**DOI:** 10.1371/journal.pone.0355938.r006

13 Jul 2026

See attached file

Attachment Submitted filename: response letter PLOS ONE revision2.docx

# Editor report: Decision Letter 3

**DOI:** 10.1371/journal.pone.0355938.r007

**Authors:** Sonia Vasconcelos

28 Jul 2026

The effect of manuscript characteristics on quality assessment of scientific manuscripts in psychology

PONE-D-24-57377R3

Dear Dr. van Assen,

We’re pleased to inform you that your manuscript has been judged scientifically suitable for publication and will be formally accepted for publication once it meets all outstanding technical requirements.

Within one week, you’ll receive an e-mail detailing the required amendments. When these have been addressed, you’ll receive a formal acceptance letter and your manuscript will be scheduled for publication.

An invoice will be generated when your article is formally accepted. Please note, if your institution has a publishing partnership with PLOS and your article meets the relevant criteria, all or part of your publication costs will be covered. Please make sure your user information is up-to-date by logging into Editorial Manager at Editorial Manager® and clicking the ‘Update My Information' link at the top of the page. For questions related to billing, please contact billing support.

If your institution or institutions have a press office, please notify them about your upcoming paper to help maximize its impact. If they’ll be preparing press materials, please inform our press team as soon as possible -- no later than 48 hours after receiving the formal acceptance. Your manuscript will remain under strict press embargo until 2 pm Eastern Time on the date of publication. For more information, please contact onepress@plos.org.

Kind regards,

Sonia Vasconcelos, PhD

Academic Editor

PLOS One

Additional Editor Comments (optional):

Reviewers' comments:

# Editor report: Acceptance letter

**DOI:** 10.1371/journal.pone.0355938.r008

**Authors:** Sonia Vasconcelos

PONE-D-24-57377R3

PLOS One

Dear Dr. van Assen,

I'm pleased to inform you that your manuscript has been deemed suitable for publication in PLOS One. Congratulations! Your manuscript is now being handed over to our production team.

At this stage, our production department will prepare your paper for publication. This includes ensuring the following:

* All references, tables, and figures are properly cited

* All relevant supporting information is included in the manuscript submission,

* There are no issues that prevent the paper from being properly typeset

You will receive further instructions from the production team, including instructions on how to review your proof when it is ready. Please keep in mind that we are working through a large volume of accepted articles, so please give us a few days to review your paper and let you know the next and final steps.

Lastly, if your institution or institutions have a press office, please let them know about your upcoming paper now to help maximize its impact. If they'll be preparing press materials, please inform our press team within the next 48 hours. Your manuscript will remain under strict press embargo until 2 pm Eastern Time on the date of publication. For more information, please contact onepress@plos.org.

You will receive an invoice from PLOS for your publication fee after your manuscript has reached the completed accept phase. If you receive an email requesting payment before acceptance or for any other service, this may be a phishing scheme. Learn how to identify phishing emails and protect your accounts at https://explore.plos.org/phishing.

If we can help with anything else, please email us at customercare@plos.org.

Thank you for submitting your work to PLOS One and supporting open access.

Kind regards,

PLOS One Editorial Office Staff

on behalf of

Dr. Sonia Vasconcelos

Academic Editor

PLOS One
